# Prevalence and predictors of artificial intelligence chatbots use for caregiving: Evidence from the Understanding America Study

**DOI:** 10.1371/journal.pone.0357753

**Published:** 2026-09-23

**Authors:** Joan K. Monin, Marco Angrisani, Tate Dunbar, Angeliki Karachaliou, Kira Birditt

**Affiliations:** 1 Social and Behavioral Sciences, Yale School of Public Health, New Haven, Connecticut, United States of America; 2 Center for Economic and Social Research, University of Southern California, Los Angeles, California United States of America; 3 Survey Research Center, Institute for Social Research, University of Michigan, Ann Arbor, Connecticut, United States of America; Anhui Polytechnic University, CHINA

## Abstract

Support for the growing population of family caregivers in the United States remains a challenging public health issue. With a limited workforce to support families coping with chronic and degenerative conditions, caregivers are turning to individualized approaches to gain information and emotional support. One approach that may be gaining popularity in caregiving support is the use of Artificial Intelligence (AI) chatbots. This study examined caregivers’ awareness and general use of AI chatbots, their use of AI chatbots for caregiving purposes, and variations in these outcomes by individual characteristics (e.g., the Big 5 personality traits, internet literacy, cognitive ability) and caregiving contextual factors (e.g., caregiving relationship, caregiving stress, the health condition of the care recipient). To this end, we used one of the waves of the Understanding America Study-Caregiving Lifecourse Experiences Assessed in Real-time (UAS-CLEAR), a longitudinal study of caregivers in the U.S. The sample consisted of 1,300 caregivers, with data collected between August and October 2025. We employed a cross-sectional, mixed-methods design, combining quantitative analysis of structured survey items with thematic coding of open-ended responses. Of the 487 respondents who reported being AI chatbot users, 25% used the technology for caregiving. Those who were more likely to use AI for caregiving were higher in openness, lower in internet literacy, lower in cognitive ability, helped with more caregiving tasks, and were more uncertain in their caregiving role. Over half (58%) used AI chatbots for information about caregiving, 34% for emotional support related to caregiving, and 37% reported other uses. Open-ended responses about individuals’ experiences using AI chatbots for caregiving also revealed that the most common reason for use was information seeking. In addition, caregivers found the chatbot support to be useful, while acknowledging some limitations of their use. Future research should examine perceptions of AI chatbots among non-users, the mechanisms linking individual traits to caregiving-related chatbot use, and the longitudinal effects of such use on caregiver well-being.

## Introduction

Family and social network members play a significant role in the care of people living with complex and chronic conditions. Yet support for caregivers remains a challenging public health issue. An estimated 63 million adults in the United States served as caregivers for people living with serious health conditions, up from 43.5 million in 2015 [1]. These numbers are expected to climb dramatically over the next 30 years due to population aging, as more caregivers need support. Although caregiving is often an act of love within a close relationship and can lead to positive outcomes, such as increased meaning and purpose in life [2], it is a stressor, often associated with physical, emotional, financial, and social burden and decreased quality of life [3].

To reduce burden, caregivers may need personalized information, training, a balance between caregiving responsibilities and personal life, as well as resources for emotion management [4]. However, barriers to these supports are abundant. Physical distance, unpredictable and busy schedules, costs, social stigma, and a lack of awareness regarding available options all contribute to caregivers not accessing informational and emotional support [5–7]. There are now many formal training programs for caregivers but, when attended, they are often one-off and standardized, which is insufficient to address the complex, heterogeneous, and ever evolving challenges of caregiving [5,6]. New technological tools, which largely circumvent location, time, and cost constraints, constitute a viable and effective way to support caregivers’ needs. Given recent advances, including those enabled by artificial intelligence (AI) chatbots, it is reasonable to consider how their implementation could assist caregivers in their multifaceted roles.

AI chatbots are computer programs that engage in human-like conversations with users [8]. Previously, chatbots were rule-based programs, producing various scripted replies after processing user input. However, recent advancements in AI have resulted in the development of AI-powered chatbots that dynamically and interactively generate responses to user inputs, providing a personalized and context-dependent experience [9]. Examples of these chatbots are ChatGPT, Microsoft Copilot, and Google Gemini, all of which have experienced increasing and widespread popularity in the past few years. From July 2023 to early 2025, the percentage of American adults using ChatGPT increased from 18% to 34%, demonstrating the rapid rise of AI chatbots and, therefore, the importance of understanding their possible societal impacts, from work to social relationships [10,11].

In the context of caregiving, AI chatbots promise to fill gaps in caregiver support and reduce caregiver burden by providing personalized information, emotional support, and companionship, among other potential benefits [12]. The primary advantages of using AI chatbots rather than or in addition to traditional resources are their constant availability, scalability, anonymity, and personalization capabilities. Additionally, most AI chatbots are inexpensive or entirely free to use [12–14]. Despite this range of merits, several serious concerns remain regarding the use of AI chatbots by caregivers. These include providing inaccurate information due to AI hallucinations, potentially dangerous medical and emotional advice, inadequate empathy, mismanagement of crisis scenarios, and privacy and security issues [15–19]. Because of the novelty of this technology, it remains unclear whether any potential reduction in caregiver burden will outweigh the perceived risks.

Current research on caregivers’ use of AI chatbots is concentrated primarily on the design and assessment of caregiving-specific chatbots, the evaluation of responses given by various chatbots to caregiving-related questions, and small-scale qualitative studies of caregiver perspectives and feedback on chatbot usage [12,20,21]. Other active areas of research include determining the ability of chatbots to provide companionship or emotional support, assessing their responses to general medical questions, and evaluating potential dangers and their impacts on users. Despite rapid advances in this field, there is limited understanding of how AI chatbots are currently used for caregiving in the general population. We bridge this gap by collecting primary data on caregivers’ awareness and adoption of AI chatbots overall, as well as their use specifically for caregiving-related purposes. We also examine how individual characteristics and caregiving contextual factors are associated with the use of AI chatbots for caregiving support. Finally, among caregivers who report using AI chatbots, we document the specific caregiving-related tasks for which they use the technology. In addition, we asked them to share their experience with using chatbots for caregiving in a brief open-ended format.

We examine individual differences and caregiving contextual factors to better understand how personal characteristics and the caregiving environment shape the use of AI chatbots for caregiving support [22]. Existing research suggests that, beyond demographic and socio-economic factors, personality traits play an important role in attitudes toward AI; for example, a recent review found that people scoring higher on agreeableness and openness tend to exhibit greater trust in AI [23]. Another study documented that, in a representative sample of adults residing in the United States, usage of AI chatbots is higher among individuals with greater openness, but lower among those with higher conscientiousness and agreeableness [24]. No prior study has examined personality traits related to AI use for caregiving purposes. Hence, we investigate heterogeneity in the use of AI chatbots for caregiving purposes not only by demographic and socio-economic characteristics, but also by personality traits. Additionally, we consider variables, such as cognitive ability and internet skills, that may drive the adoption of emerging technologies like AI chatbots. To our knowledge, no research has linked the use of AI chatbots for caregiving support to caregivers’ cognitive functioning or internet literacy. There are also no studies on caregiving situational variables (type of disease or disability of the care recipient, perceived strain, number of caregiving tasks) and AI chatbot use, a relationship that we can investigate and shed light on with the data at our disposal.

## Materials and methods

### The Understanding America Study

For this study, we used data from the Understanding America Study (UAS), which is a probability-based Internet panel housed at the Center for Economic and Social Research at the University of Southern California (USC) [25]. The UAS serves as a social and health sciences infrastructure for collecting data on the daily lives of families and individuals in the U.S. The UAS panel currently comprises about 14,000 individuals aged 18 and older and is expected to grow to 20,000 respondents by 2028. Within the UAS, the authors of this paper lead a project called the “UAS-Caregiving Lifecourse Experiences Assessed in Real-time (UAS-CLEAR).” Through this project, we added longitudinal modules to the UAS to capture the experience and well-being of caregivers. These questionnaires also included items eliciting how, and to what extent, caregivers engage with AI chatbots in general and to support their caregiving roles and responsibilities more specifically.

### Research design

The current study was a cross-sectional, observational, mixed-methods analysis based on one of the waves of the UAS-CLEAR longitudinal study, namely survey UAS743, collected between August and October 2025. The quantitative component of the analysis used structured survey data on individual characteristics, caregiving contextual factors, and caregivers’ use of AI chatbots for caregiving purposes. The qualitative component relied on open-ended responses describing caregivers’ experiences with AI chatbots.

### Participants

The UAS-CLEAR is a longitudinal project that follows a sample of caregivers, drawn from a probability-based population-representative panel, over time. The first UAS-CLEAR survey (UAS634), administered between September and December 2024, identified about 2,200 caregivers. Conditional on remaining caregivers, this initial cohort was subsequently interviewed between February and June 2025 (UAS667) and again between August and October 2025 (UAS743). For the purposes of this study, we considered only respondents who, at the time survey UAS743 was administered, answered “yes” to the following question: “In a recent survey, you told us that you were assisting someone (parent, grandparent, wife, husband, child, another family member, neighbor, or close friend) with basic personal activities because they cannot handle them without help. By assisting someone with basic personal activities, we mean daily activities such as dressing, eating, bathing, paying bills, managing medication, food preparation, grocery shopping, doctor visits, emotional support, driving, and other personal assistance. Please exclude assistance given to children who are not yet self-sufficient due to their age (for example, too young to dress themselves or unable to prepare a meal). Is this still the case? Note that this can include a person you mentioned previously or a new person you are helping.”

A total of 1,409 individuals reported being caregivers in UAS743. After excluding respondents with missing values in either the outcome variables of interest or covariates, the analytic sample consisted of 1,300 caregivers. Table 1 reports the demographic characteristics of the analytic sample. Women were overrepresented, comprising 68% of caregivers. The age distribution was relatively balanced: 17% of caregivers were aged 18–39, 19% were aged 40–49, 22% were aged 50–59, 24% were aged 60–69, and 17% were aged 70 or older. Non-Hispanic White caregivers accounted for 57% of the sample, while non-Hispanic Black and non-Hispanic Other caregivers each represented approximately 13%; Hispanic caregivers comprised 16% of the sample. Approximately one-quarter of respondents had a high school education or less, while the remainder reported some college education (38%) or a college degree or higher (38%). Slightly more than half of the sample (55%) were married or partnered, 21% were separated, divorced, or widowed, and 24% had never married. Household income was evenly distributed across categories, with roughly one-quarter of respondents in each income group. About two-thirds of caregivers resided in the South or West, while 15% and 19% lived in the Northeast and Midwest, respectively. Finally, approximately half of the sample was employed, with the remainder retired or otherwise out of the labor force.

**Table 1:** Analytic sample’s demographic composition and individual traits.

	Mean	Standard Deviation	Range
**Demographics**			
Gender: Male	0.315	0.465	0-1
Gender: Female	0.685	0.465	0-1
Age 18–39	0.173	0.378	0-1
Age 40–49	0.194	0.395	0-1
Age 50–59	0.221	0.415	0-1
Age 60–69	0.238	0.426	0-1
Age 70+	0.174	0.380	0-1
Race/Ethnicity: non-Hispanic White	0.575	0.494	0-1
Race/Ethnicity: non-Hispanic Black	0.133	0.339	0-1
Race/Ethnicity: non-Hispanic Other	0.134	0.341	0-1
Race/Ethnicity: Hispanic	0.158	0.365	0-1
Education: High school or less	0.231	0.422	0-1
Education: Some college	0.385	0.487	0-1
Education: College or more	0.384	0.486	0-1
Marital Status: Married	0.555	0.497	0-1
Marital Status: Separated/Divorced/Widowed	0.209	0.407	0-1
Marital Status: Never married	0.236	0.424	0-1
Household Income: < $30,000	0.255	0.436	0-1
Household Income: $30,000-$59,999	0.242	0.429	0-1
Household Income: $60,000-$99,999	0.241	0.428	0-1
Household Income: >=$100,000	0.262	0.440	0-1
Census Region: Northeast	0.147	0.354	0-1
Census Region: Midwest	0.192	0.394	0-1
Census Region: South	0.323	0.468	0-1
Census Region: West	0.338	0.473	0-1
Labor Force Status: Working	0.509	0.500	0-1
Labor Force Status: non-Working	0.491	0.500	0-1
**Individual Traits**			
Agreeableness	35.977	5.622	9-45
Conscientiousness	35.725	5.818	9-45
Extroversion	25.155	6.374	8-40
Neuroticism	22.200	6.703	8-40
Openness	35.962	6.099	10-50
Cognitive Ability Score	151.975	22.815	70-202
Internet Literacy	12.832	4.412	0-17

Table 1 also reports summary statistics for individual traits in the analytic sample. Mean scores and standard deviations indicate substantial heterogeneity across all Big-5 personality traits, which were measured following standard methodologies. In our analytic sample, Cronbach’s alpha values indicated acceptable to high internal consistency for all five personality dimensions: Openness α = 0.78; Conscientiousness α = 0.81; Extroversion α = 0.82; Agreeableness α = 0.77; Neuroticism α = 0.84 (additional details about how the Big-5 personality traits are measured can be found on the UAS website: https://uasdata.usc.edu/survey/ UAS + 593) [26,27]. Because these traits were measured on different scales, comparisons of average levels across traits should be interpreted with caution. To provide additional context, mean scores corresponded to approximately 55–80% of each trait’s maximum possible value, suggesting meaningful variation within each personality dimension. Cognitive ability was measured as the sum of scores from three components of the Woodcock–Johnson Tests of Cognitive Abilities [28]: quantitative reasoning (number series) and lexical knowledge (picture vocabulary and verbal analogies). Each component score was converted into a T-score, with a mean of 50 and a standard deviation of 10 in the general U.S. population (full details about the scoring methodology can be found on the UAS website: https://uasdata.usc.edu/page/ Cognitive+Comprehensive+File). In the analytic sample, the average total cognitive ability score was 152, consistent with population norms. Cronbach’s alpha for the composite cognitive-ability score was 0.77, indicating acceptable internal consistency. Internet literacy was measured as the sum of 17 dichotomous indicators capturing familiarity with basic internet concepts and tasks (e.g., browsing, email, file management); none of these items reference AI tools, AI chatbots, or related technologies, so internet literacy is conceptually distinct from awareness and use of AI chatbots, which are the outcomes of interest in this study. In our analytic sample, the average internet literacy score was 12.8, indicating moderate to relatively high levels of internet literacy among sampled caregivers; Cronbach’s alpha for the internet literacy index was 0.90, suggesting excellent internal consistency across the items used to construct the index (additional details can be found on the UAS website: https://uasdata.usc.edu/page/Comprehensive+ File+And+Panel+Dataset).

### Caregiving-related measures

Our survey (UAS743) contained a comprehensive set of questions designed to capture caregiving responsibilities, tasks, circumstances, and associated well-being outcomes. We used these data to construct measures of caregiving intensity and perceived burden (the full UAS743 questionnaire is available on the UAS website: https://uasdata.usc.edu/survey/UAS+743). Caregivers were asked to report the number of individuals they regularly assist, their relationship with the care recipient(s), and the care recipient(s)’ living arrangements. As shown in Table 2, most respondents (78%) provided care to a single individual. The most common care-recipient relationship was a parent (44%), followed by other relatives or non-relatives (24%), a spouse or partner (20%), and a son or daughter (12%). The sample was evenly split between caregivers who co-resided with the care recipient and those whose care recipient lived elsewhere. Respondents also reported all health conditions experienced by their care recipient(s). We group these conditions into three categories: (1) cognitive and neurodegenerative conditions, including Alzheimer’s disease or ADRD, intellectual disability, and mental health disorders, reported by 41% of caregivers; (2) physical disability and functional impairment, including physical disability, stroke, frailty, vision impairment, and age-related impairments, reported by approximately 60% of caregivers; and (3) chronic medical conditions, including diabetes, heart disease, cancer, other chronic illnesses, and long-term COVID, reported by 42% of caregivers.

**Table 2:** Caregiving-related variables.

	Mean	Standard Deviation	Range
Multiple CRs: No	0.776	0.417	0-1
Multiple CRs: Yes	0.224	0.417	0-1
CR’s Relationship: Spouse/Partner	0.205	0.403	0-1
CR’s Relationship: Parent	0.435	0.496	0-1
CR’s Relationship: Son/Daughter	0.118	0.322	0-1
CR’s Relationship: Other	0.243	0.429	0-1
CR’s Lives with Caregiver	0.498	0.500	0-1
CR’s Lives Elsewhere	0.502	0.500	0-1
CR’s Condition: Cognitive/Neurodegenerative (No)	0.585	0.493	0-1
CR’s Condition: Cognitive/Neurodegenerative (Yes)	0.415	0.493	0-1
CR’s Condition: Physical Disability/Impairment (No)	0.405	0.491	0-1
CR’s Condition: Physical Disability/Impairment (Yes)	0.595	0.491	0-1
CR’s Condition: Chronic Illness (No)	0.577	0.494	0-1
CR’s Condition: Chronic Illness (Yes)	0.423	0.494	0-1
Help Intensity Score	6.014	2.299	0-10
Lack of Time	2.295	1.060	1-5
Feeling Stressed	2.451	1.064	1-5
Feeling Strained	2.309	1.043	1-5
Feeling Uncertain	2.148	1.043	1-5

Note. CR = care recipient.

To measure caregiving intensity, caregivers indicated whether they assisted the care recipient with each of ten tasks, including household chores, shopping, transportation, financial management, personal care, mobility assistance, medication management, use of medical equipment, healthcare coordination, and emotional support. For each task performed by the caregiver, we constructed a binary indicator and summed these indicators to create a help-intensity score ranging from 0 to 10. On average, caregivers in the sample reported assisting with approximately six of the ten tasks. Finally, perceived caregiving burden was measured using four self-reported variables capturing how frequently caregivers felt they lacked time for themselves, experienced stress balancing caregiving with other responsibilities, felt strained when around the care recipient, and felt uncertain about their caregiving role. Each outcome was measured on a five-point Likert scale ranging from “never” to “nearly always.” As reported in Table 2, mean values for these measures were only slightly above 2, indicating relatively modest average impacts of caregiving on caregivers’ well-being.

### Outcome variables

Our outcome variables were intended to capture caregivers’ awareness and use of AI chatbots, both overall and specifically for caregiving purposes. To measure awareness and general use of AI applications, the survey asked respondents the following question:

“AI refers to the ability of a computer or machine to perform tasks that typically require human intelligence, such as learning from experience, solving problems, and making decisions. Recently developed AI applications, such as ChatGPT, Llama, Gemini, Grok, and Copilot, can provide information, companionship, conversation, and sometimes even emotional support to people. They can be like a ‘digital human,’ available to chat, listen, and interact. They can also help caregivers manage caregiving tasks. Have you heard about or used AI applications such as ChatGPT, Llama, Gemini, Grok, or Copilot?” Respondents could select one of the following response options: “I have never heard about them,” “I have heard about them but I have never used one,” “I have used them,” and “I don’t know.”

Respondents who indicated that they had used an AI chatbot were then asked: “Have you used AI applications such as ChatGPT, Llama, Gemini, Grok, or Copilot to assist you in your caregiving role?”

Respondents who answered this question affirmatively were asked: “What have you used these AI applications for as a caregiver? Please select all that apply (Responses: – For information about caregiving; – For emotional support about caregiving; – Other, please specify). In addition, these respondents were invited to briefly describe, in a few sentences, their experiences using AI applications such as ChatGPT, Llama, Gemini, Grok, or Copilot to assist them in their caregiving role.

### Statistical analysis

We first conducted descriptive analyses, reporting frequencies to characterize caregivers’ awareness of AI chatbots, overall use of this technology, and use specifically for caregiving purposes. We also computed frequencies to describe the caregiving-related activities for which AI chatbots were used.

We then estimated a logistic regression model predicting, among the subsample of caregivers who reported using AI chatbot in general (n = 487), predicting AI chatbot use for caregiving, specifically. The model included covariates capturing demographic characteristics – sex, age, marital status, education, race/ethnicity, work status, household income, and Census region – and individual traits – cognitive ability, internet literacy, and the Big-5 personality traits (openness, conscientiousness, extroversion, agreeableness, and neuroticism). In terms of caregiving contextual factors, we controlled for whether the caregiver assisted more than one person, the care recipient’s relationship to the caregiver, conditions, and living arrangements. We also included the caregiving help intensity score and measures of caregiving burden – lack of time, stress related to balancing caregiving and other responsibilities, feelings of strain, and uncertainty about caregiving.

For the Logit regression model, the dependent variable was an indicator equal to 1 if the respondent reported using chatbots for caregiving. We estimated three nested specifications, with results reported in Table 4: a baseline model including only demographic characteristics (column 1), a second specification that added individual traits (column 2), and a third specification that further incorporated caregiving-related variables (column 3). Our richer specifications included several conceptually related covariates, notably two measures of individual cognitive and digital skills (cognitive ability and internet literacy) and four self-reported measures of caregiving burden (lack of time, stress balancing caregiving with other responsibilities, feelings of strain, and uncertainty about the caregiving role). We retained these variables jointly because they relate to theoretically distinct constructs. Specifically, cognitive ability captures general intellectual functioning, while internet literacy measures digital skills, which are more proximate to engagement with a chatbot interface; the four burden items reflect, respectively, temporal pressure, role-conflict stress, relational strain, and role-efficacy uncertainty. To verify that collinearity does not threaten the stability of our estimates, we computed variance inflation factors (VIFs) for all covariates in the most saturated specification (column 3 of Table 4). The maximum VIF was 3.07, and the mean VIF was 1.83, both well below the conventional threshold of 5.

Results are reported as marginal effects from Logit models and interpreted as percentage-point changes in the probability of using AI chatbot for caregiving. To ease interpretation, continuous scores for individual traits – personality traits, cognitive ability, and internet literacy – were included in the regressions as z-scores, with a sample mean of 0 and a standard deviation of 1. In Supporting Information, we report the estimated marginal effects reflecting the effect of demographic characteristics, individual traits, and caregiving-related variables on the likelihood of using AI chatbots in general, not for caregiving purposes specifically. All models were estimated using heteroskedasticity-robust standard errors.

For the analysis of the open-ended responses to the question capturing caregivers experiences using AI chatbots for caregiving, one coder (JM) read all of response (n = 128) and generated the following themes: “informational support”, “emotional support”, “AI chatbot is useful”, “AI chatbot has limitations”, “help with language/writing”, “type of AI chatbot mentioned”, “Did not use for caregiving”, and “Unable to code”. Two independent raters (TD and AK) associated the responses with one of these non-mutually exclusive themes, separately. We determined interrater reliability by the percentage of agreement of all three ratings (100% agreement was achieved when all coders associated the same response with the same theme). See Table 3 for the mean agreement for each theme that required rating. The first rater’s responses were used to generate frequencies of each theme in the analysis.

**Table 3:** Interrater reliability of the themes.

Theme	Mean coder agreement on responses (0–100%)
Informational support	93%
Emotional support	97%
AI chatbot is useful	83%
AI chatbot has limitations	93%
Help with language/writing	89%
Not able to code	82%

## Results and discussion

### What percentage of the sample said they were aware of AI chatbots and used them?

**Fig 1:**
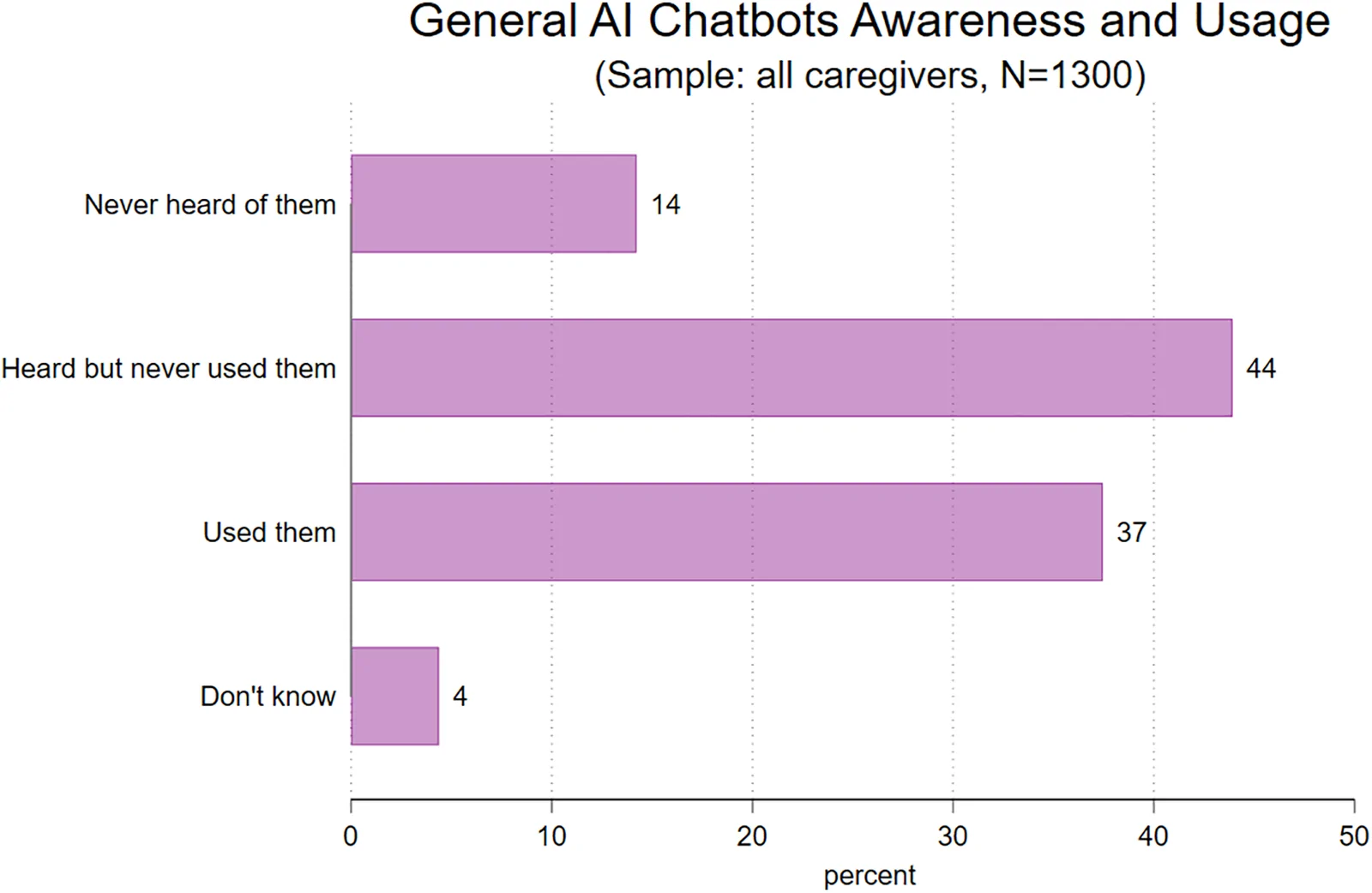
General AI chatbots awareness and usage (sample: all caregivers, N = 1300).

As shown in Fig 1, most caregivers heard of AI chatbots but did not use them (44%, n = 571), while 37% (n = 487) used them and 14% (n = 185) never heard of them, with the remaining 4% (n = 57) answering “don’t know” (percentages do not sum to 100 due to rounding).

### What percentage of the AI chatbot users used them for caregiving and what did they use them for related to caregiving?

**Fig 2:**
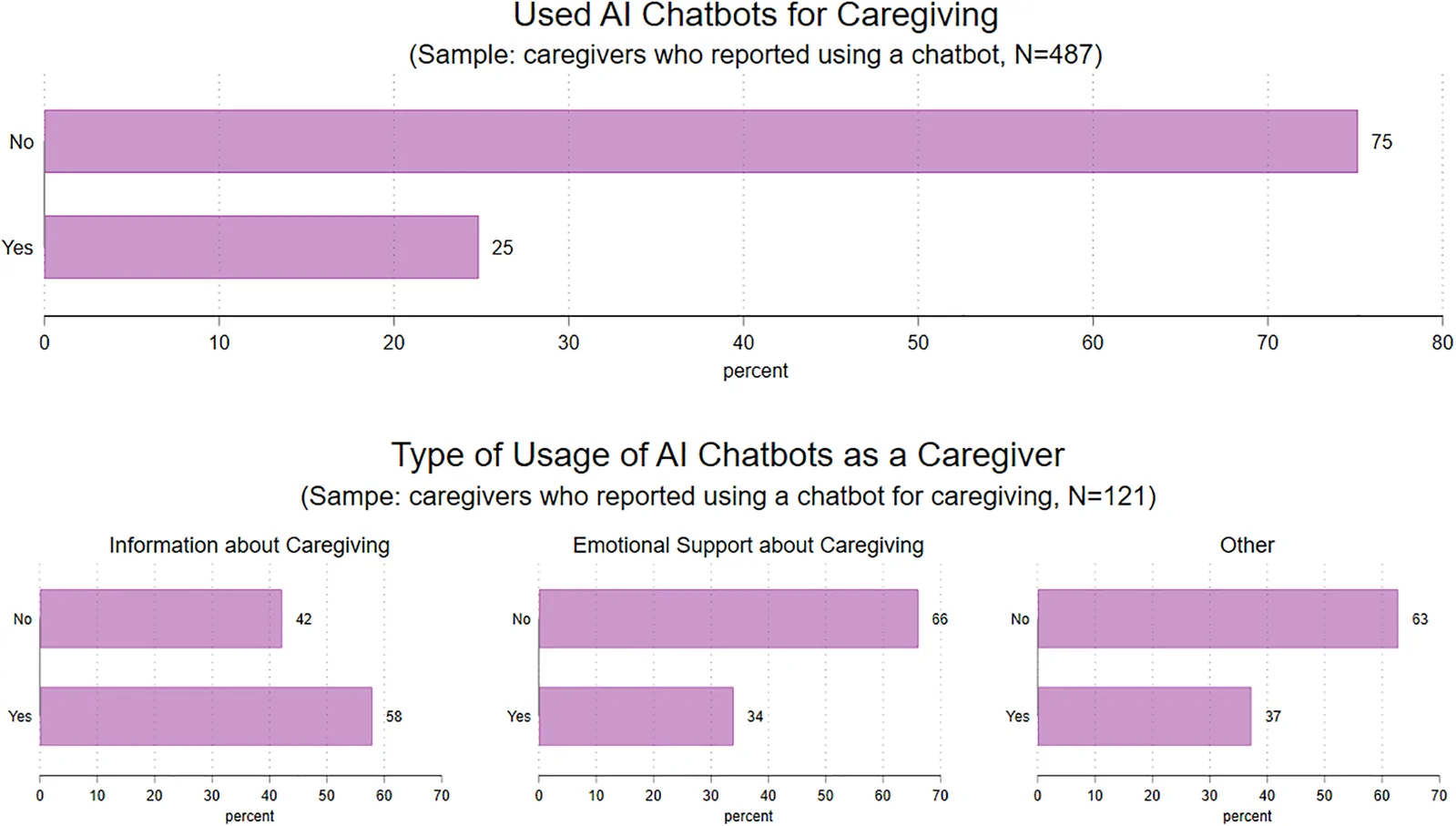
(a) Used AI Chatbots for Caregiving (Sample: caregivers who reported using a chatbot, N = 487); (b) Type of Usage of AI Chatbots as a Caregiver (Sample: caregivers who reported using a chatbot for caregiving, N = 121).

As shown in Fig 2, 25% (n = 121 out of 487) of caregivers who used AI chatbots said they used them for caregiving. These respondents reported using this technology mostly for informational support (58%, n = 70), with emotional support and other reasons receiving endorsement rates of 34% (n = 41) and 37% (n = 45), respectively. We categorized the write-in responses according to the response options from the close-ended question: informational support, emotional support, and other. Out of the 45 “write-in” responses, 24 referred to informational support, 1 to emotional support, and 19 to other reasons. The latter category mostly included using AI for other purposes besides caregiving (e.g., work-related tasks). Creative purposes, such as searching for activities to do with their care recipient and for music, were mentioned by two respondents.

### How individual characteristics and caregiving-related variables predict AI chatbot use for caregiving purposes

Table 4 presents the results of regressions, estimated on the subsample of caregivers who report having used AI chatbots in general (n = 487), predicting the likelihood of using AI chatbots specifically for caregiving purposes. Within this group, demographic characteristics were generally not significantly associated with the likelihood of using AI chatbots for caregiving, with two exceptions: caregivers who have never married and those residing in the South exhibited significantly lower probabilities of AI chatbot use for caregiving. Consistent with the patterns observed for general chatbot use (see S1Table), a one-standard deviation increase in openness was associated with a 4–5 percentage point increase in the likelihood of using AI chatbots for caregiving support. In contrast, caregivers with higher cognitive ability and greater internet literacy were significantly less likely to rely on AI chatbots for caregiving assistance. With respect to caregiving-related characteristics, we found suggestive evidence that caregivers who assist with a larger number of tasks were more likely to use AI chatbots for caregiving, although this association was only marginally statistically significant. Notably, greater self-reported uncertainty about how to care for the care recipient was associated with a higher likelihood of using AI chatbots for caregiving support.

**Table 4:** Likelihood of using AI chatbots for caregiving.

	(1)	(2)	(3)
VARIABLES	y1	y1	y1
Male	−0.008	−0.001	0.009
	(0.042)	(0.045)	(0.045)
Age 40–49	−0.097	−0.065	−0.057
	(0.059)	(0.057)	(0.054)
Age 50–59	−0.073	−0.037	−0.012
	(0.063)	(0.063)	(0.061)
Age 60–69	−0.053	−0.028	0.003
	(0.071)	(0.075)	(0.071)
Age 70+	−0.101	−0.117	−0.072
	(0.091)	(0.086)	(0.096)
Black	0.024	−0.019	−0.016
	(0.067)	(0.062)	(0.063)
Other	0.065	0.045	0.044
	(0.058)	(0.061)	(0.062)
Hispanic	0.069	0.038	0.038
	(0.064)	(0.059)	(0.057)
Some college	−0.085	−0.056	−0.075
	(0.065)	(0.059)	(0.059)
College degree or more	−0.071	−0.019	−0.051
	(0.066)	(0.064)	(0.065)
Sep/Div/Wid	−0.007	0.002	−0.021
	(0.059)	(0.058)	(0.056)
Never married	−0.085*	−0.086*	−0.083*
	(0.046)	(0.046)	(0.047)
Hinc $30k-$60k	0.046	0.063	0.061
	(0.066)	(0.059)	(0.057)
Hinc $60k-$100k	0.020	0.079	0.068
	(0.069)	(0.065)	(0.064)
Hinc $100k+	−0.027	0.053	0.045
	(0.067)	(0.066)	(0.067)
Working	−0.020	−0.021	−0.010
	(0.046)	(0.045)	(0.044)
Midwest	−0.134*	−0.097	−0.115
	(0.078)	(0.076)	(0.074)
South	−0.146**	−0.138**	−0.167***
	(0.068)	(0.064)	(0.063)
West	−0.105	−0.099	−0.113*
	(0.070)	(0.066)	(0.064)
Agreeableness		−0.034*	−0.028
		(0.020)	(0.020)
Conscientiousness		−0.011	−0.007
		(0.022)	(0.022)
Extroversion		0.022	0.023
		(0.020)	(0.020)
Neuroticism		0.012	0.011
		(0.024)	(0.024)
Openness		0.059***	0.063***
		(0.020)	(0.021)
Cognitive ability		−0.061**	−0.060**
		(0.024)	(0.025)
Internet literacy		−0.078***	−0.074***
		(0.026)	(0.026)
Multiple care recipients			0.071
			(0.045)
CR: parent			0.054
			(0.061)
CR: son/daughter			0.088
			(0.083)
CR: other			0.068
			(0.076)
CR lives elsewhere			0.030
			(0.047)
CR’s cond: cognitive /neurodegenerative			0.058
			(0.042)
CR’s cond: physical disability/impairment			0.027
			(0.040)
CR’s cond: chronic illness			0.001
			(0.041)
Help intensity score			0.019*
			(0.010)
Lack of time			−0.016
			(0.026)
Stressed			−0.002
			(0.029)
Strained			−0.038
			(0.027)
Uncertain			0.062***
			(0.023)
Observations	487	487	487

Note: Sample restricted to caregivers who reported general AI chatbot use (n = 487); the dependent variable indicates use of AI chatbots specifically for caregiving purposes. Heteroskedasticity-robust standard errors in parentheses. *** p < 0.01, ** p < 0.05, * p < 0.1.

In terms of the open-ended responses describing individuals’ experiences using AI applications for caregiving purposes, Table 5 shows that the most common theme was seeking informational support (72 out of 128, 56%), whereas only 9 (7%) provided description consistent with AI chatbots being used for emotional support. Forty-eight (38%) responses referred to how an AI chatbot is useful for caregiving purposes. For example, people described it as “quick and easy” or stated, “Realizing that it is not always 100% correct, I find it very useful and helpful and amazing”, or reported “I found it extremely helpful when it came to information gathering” and “It was also very efficient at giving a condensed summary of a continuous article about caregiving”. Twelve (9%) responses explicitly indicated that AI chatbots had limitations. For example, respondents wrote “the chatGPT was kind of useless i had to say the same question over and that was irratating” and “not where it needs to be yet.” Regarding the types of chatbots mentioned in the open-ended responses, we found 31 (24%) mentions of ChatGPT, 2 (1.5%) of Gemini, and 1 (<1%) of Copilot. Help with language and writing was reported 8 times (6%) as being a use for caregivers.

**Table 5:** Frequencies of themes in open ended responses (n = 128).

Theme	N (%)	Example response
Informational support	72 (56%)	“it helps with basic questions. of course major medical issues I wouldnt allow it to advise”
Emotional support	9 (7%)	“ChatGPT is very helpful and understanding. It gives me support that I'm doing the right thing with my mom. It also gives me information locally that could help.”
AI chatbot is useful	48 (38%)	“me explica muy bien lo que debo hacer para el cuidado” [He/She explains very well to me what I should do for the care]
AI chatbot has limitations	12 (9%)	“sometimes it's nicely written, sometimes its very sloppy, self repetitive, and self-contradicting too!”
Help with language/writing	8 (6%)	“… i've used it to write documents”
Type of chatbot mentioned in response	34 (27%)	“ChatGPT, I was looking for community resources to help pay for my daughter's [medical] bills that are related to her [condition].”
Not able to code	12 (9%)	N/A

Note. Not mutually exclusive themes. Two participants said they did not use for caregiving.

## Conclusions

This study provides a snapshot of the extent to which caregivers are aware of and use AI chatbots for caregiving purposes and the individual characteristics and caregiving contextual factors that relate to their use. We found that 25% of the caregivers who use AI chatbots in general also use them in their caregiving role, primarily for information seeking. Some also reported using AI chatbots for emotional support. Individual characteristics and traits played a role in the likelihood of using AI chatbots for caregiving purposes.

Our findings align with other studies showing that people high in openness are more likely to trust AI [23,24]. In addition, we found that those with lower cognitive abilities and internet literacy were more likely to use AI chatbots for caregiving, a pattern that we interpret with caution, given the cross-sectional and observational nature of our data. One plausible mechanism, consistent with open-ended responses describing chatbots as “quick and easy” to use, is that AI chatbots provide an accessible information channel for caregivers who may find it harder to navigate other sources (e.g., medical literature, healthcare professionals, or community-based programs). However, several alternative mechanisms are equally compatible with the observed associations. First, caregivers with lower cognitive ability or internet literacy may be less able to identify the limitations of chatbot output, leaving them more reliant on chatbots without seeking corroboration; conversely, more digitally literate caregivers may be more aware of these risks and deliberately avoid chatbots for caregiving-related decisions. Second, chatbots may substitute for other forms of support, such as professional consultation or peer communities, that less digitally literate caregivers find harder or more costly to access. Third, the observed pattern may partly reflect unobserved characteristics that jointly shape cognitive functioning, internet literacy, and the propensity to engage with novel tools. We could not distinguish between these mechanisms with the data at our disposal. We also note that the identification of the most plausible mechanism(s) is unlikely to be achievable through additional waves of survey data alone, since cognitive ability and internet literacy are largely stable individual characteristics that change slowly, if at all, over the short and medium term.

Our findings also extend beyond individual differences and use of AI by examining them alongside other caregiving situational factors. It was not surprising that when caregivers reported being more uncertain in their role, they were more likely to say they used AI chatbots for caregiving. When people are uncertain, they are more likely to seek information, the primary use of chatbots in our sample [29]. It was unexpected, however, that other factors like care recipient disease type, stress, and lack of time were not associated with AI chatbot use for caregiving purposes.

The qualitative analysis of open-ended responses pointed to a few research directions to pursue in the future. Many caregivers’ responses about their experiences with AI chatbots noted that the chatbots are quick, easy, and helpful, suggesting they were largely satisfied with the information they obtained this way. However, some did offer caveats about using AI chatbots, noting that one needs to know how to use them correctly. For example, caregivers who use AI chatbots for advice about cures or treatments for their care recipient may be vulnerable to misinformation, especially if they are unable to verify it. Future research could address this by helping caregivers understand which information is accurate and which requires follow-up by a third party or a professional, such as a medical doctor or nurse.

Strengths of the present study are the primary collection of data about AI chatbot usage in a demographically diverse sample of caregivers, drawn from a probability-based, population-representative panel, and the variety of individual-level characteristics – demographics, socio-economic factors, and traits – as well as caregiving-related variables – care recipient age, relationship types, and caregiving intensity – available for each survey participant. A limitation was that we had budgetary and time constraints with the survey design that made it only possible to ask for brief responses for our qualitative analysis. However, we see this as a starting point for hypothesis generating for future quantitative studies. That said, the qualitative analysis was affected by the brevity of responses, and the coding process was limited by the bounds of using this data source. The initial development of coding themes by a single coder and the use of percent agreement as the reliability measure was appropriate for exploratory analysis, but further work is needed to take more rigorous approaches to identifying themes either using inductive approaches or top-down approaches guided by theory. Although rater agreement was high, biases may be present from the one initial coder generating the themes. We recommend that subsequent studies utilize independent double-blind thematic coding from inception, instead of post-hoc verification, to ensure psychometric validity and inductive objectivity.

A second potential limitation concerns how we operationalized caregivers’ use of AI chatbots for caregiving purposes. The question eliciting this key measure intentionally relied on respondents’ own judgment of what counts as caregiving-related use, rather than imposing a narrow functional criterion. The examination of open-ended responses indicates that a subset of respondents who reported using chatbots for caregiving purposes described uses that were only indirectly related to caregiving, or, in some cases, unrelated to it (e.g., assistance with work tasks, creative purposes, or general information-seeking). We adopted this self-definition approach deliberately: caregiving research commonly relies on respondents’ self-identification of their caregiving role and responsibilities. In an early descriptive study of an emerging behavior, a narrower functional definition risked excluding novel or context-dependent uses that caregivers themselves consider relevant to their role. We acknowledge, however, that this choice introduces some ambiguity. Our prevalence estimates of caregiving-related AI chatbot use should therefore be interpreted as reflecting caregivers’ own judgments about what qualifies as caregiving use, rather than a narrowly defined functional criterion imposed by the researchers.

A future direction generated by these findings is to understand caregivers’ perceptions about the use of AI chatbots for caregiving among a sample that includes both users and non-users of AI chatbots. Those who use AI chatbots differ in many ways from the population of caregivers (as shown by our results in S1Table). For example, younger and more affluent individuals have more access to AI chatbots and may have a better understanding of how they can be used in their caregiving role. For those without prior experience, using vignettes that talk about what AI chatbots can do and how they would want to use them may be beneficial, if access can also be provided. A further development is to disentangle the mechanisms underlying the observed negative associations between AI chatbot use for caregiving and both cognitive ability and internet literacy. To this end, future data collection should include richer measures of the intermediate constructs that plausibly link these individual traits to caregiving behavior, such as caregivers’ perceived accuracy of chatbot output, their trust in alternative information sources, and their awareness of chatbot limitations. Another future direction of this research is to examine whether the use of chatbots for caregiving is associated with improvements in well-being over time as well as to identify individual and contextual factors that predict for whom they are more or less useful in improving well-being. For example, is the informational support from AI chatbots enough to improve caregiving outcomes? Are there profiles (that consist of individual and caregiving contextual factors) of caregivers who want emotional support from chatbots in the larger population, and how would this use influence their well-being?

In conclusion, the intention of this study was to determine whether caregivers are currently using widely accessible AI chatbots. While caregiving specific AI chatbots are being developed and on the cusp of being implemented, we provide information about how general AI chatbots are being used by caregivers. In general, we found that relatively few caregivers are using widely available AI chatbots for caregiving purposes. Greater use of AI chatbots for caregiving is related to a person’s greater openness to experience, decreases with the level of internet literacy and cognitive ability, and increases with the extent to which caregivers feel uncertain in their caregiving role. Overall, caregivers mainly wanted to use the AI chatbots for information-seeking. Our empirical findings highlight key considerations for translating AI chatbot technologies into effective supports for caregivers.

## Supporting information

S1 Table How individual characteristics and caregiving-related variables predict AI chatbot use in general. (DOCX)
