# Supplementary material for: Prevalence and predictors of artificial intelligence chatbots use for caregiving: Evidence from the Understanding America Study
Source: PLoS One. 2026 Sep 23;21(9):e0357753. doi: 10.1371/journal.pone.0357753 (PMC13600484; doi:10.1371/journal.pone.0357753)
Supplement: Supplementary file 1 [file pone.0357753.s001.docx]

**Supporting Information**

**How individual characteristics and caregiving-related variables predict AI chatbot use in general**

As shown in the table below, the likelihood of using AI-driven chatbots in general was higher among younger caregivers, those with a college degree, and those with annual household income of $100,000 or more. With respect to individual traits, a one-standard deviation increase in openness was associated with an approximately four-percentage point increase in the probability of AI chatbot use, while a one-standard deviation increase in extroversion was associated with about a two-percentage point increase (although this coefficient was only marginally significant at 10%). Caregivers with higher levels of internet literacy, which may proxy for broader digital skills, were substantially more likely to use AI chatbots. In contrast, none of the caregiving-related variables were significantly associated with general AI chatbot use.

Likelihood of Using AI Chatbots in General

|  | (1) | (2) | (3) |
| --- | --- | --- | --- |
| VARIABLES | y1 | y1 | y1 |
|  |  |  |  |
| Male | 0.012 | 0.019 | 0.025 |
|  | (0.028) | (0.028) | (0.028) |
| Age 40-49 | -0.038 | -0.042 | -0.042 |
|  | (0.044) | (0.042) | (0.042) |
| Age 50-59 | -0.144*** | -0.144*** | -0.149*** |
|  | (0.044) | (0.044) | (0.044) |
| Age 60-69 | -0.263*** | -0.234*** | -0.226*** |
|  | (0.045) | (0.046) | (0.047) |
| Age 70+ | -0.395*** | -0.351*** | -0.327*** |
|  | (0.046) | (0.050) | (0.054) |
| Black | -0.040 | -0.012 | -0.012 |
|  | (0.039) | (0.042) | (0.042) |
| Other | -0.004 | 0.029 | 0.023 |
|  | (0.038) | (0.039) | (0.038) |
| Hispanic | -0.037 | -0.027 | -0.031 |
|  | (0.037) | (0.037) | (0.037) |
| Some college | 0.083** | 0.052 | 0.051 |
|  | (0.032) | (0.034) | (0.034) |
| College degree or more | 0.265*** | 0.210*** | 0.203*** |
|  | (0.035) | (0.037) | (0.038) |
| Sep/Div/Wid | 0.009 | -0.002 | -0.007 |
|  | (0.035) | (0.034) | (0.035) |
| Never married | -0.000 | -0.017 | -0.016 |
|  | (0.033) | (0.032) | (0.033) |
| Hinc $30k-$60k | 0.064* | 0.040 | 0.036 |
|  | (0.037) | (0.038) | (0.039) |
| Hinc $60k-$100k | 0.043 | 0.005 | 0.003 |
|  | (0.040) | (0.040) | (0.041) |
| Hinc $100k+ | 0.138*** | 0.090** | 0.080* |
|  | (0.043) | (0.044) | (0.045) |
| Working | 0.032 | 0.042 | 0.037 |
|  | (0.029) | (0.028) | (0.029) |
| Midwest | -0.042 | -0.027 | -0.022 |
|  | (0.043) | (0.042) | (0.041) |
| South | 0.039 | 0.043 | 0.050 |
|  | (0.039) | (0.038) | (0.037) |
| West | 0.060 | 0.063* | 0.067* |
|  | (0.039) | (0.038) | (0.038) |
| Agreeableness |  | -0.006 | -0.008 |
|  |  | (0.015) | (0.015) |
| Conscientiousness |  | 0.006 | 0.007 |
|  |  | (0.015) | (0.015) |
| Extroversion |  | 0.024* | 0.023* |
|  |  | (0.013) | (0.013) |
| Neuroticism |  | 0.036** | 0.034** |
|  |  | (0.015) | (0.016) |
| Openness |  | 0.036*** | 0.037*** |
|  |  | (0.013) | (0.013) |
| Cognitive ability |  | 0.004 | 0.006 |
|  |  | (0.016) | (0.016) |
| Internet literacy |  | 0.083*** | 0.087*** |
|  |  | (0.016) | (0.016) |
| Multiple care recipients |  |  | 0.033 |
|  |  |  | (0.029) |
| CR: parent |  |  | 0.043 |
|  |  |  | (0.044) |
| CR: son/daughter |  |  | 0.035 |
|  |  |  | (0.048) |
| CR: other |  |  | 0.010 |
|  |  |  | (0.046) |
| CR lives elsewhere |  |  | -0.035 |
|  |  |  | (0.031) |
| CR’s cond: cognitive /neurodegenerative |  |  | -0.026 |
|  |  |  | (0.026) |
| CR’s cond: physical disability/impairment |  |  | -0.032 |
|  |  |  | (0.027) |
| CR’s cond: chronic illness |  |  | -0.019 |
|  |  |  | (0.025) |
| Help intensity score |  |  | 0.000 |
|  |  |  | (0.006) |
| Lack of time |  |  | -0.014 |
|  |  |  | (0.018) |
| Stressed |  |  | 0.031 |
|  |  |  | (0.019) |
| Strained |  |  | 0.004 |
|  |  |  | (0.016) |
| Uncertain |  |  | -0.006 |
|  |  |  | (0.015) |
|  |  |  |  |
| Observations | 1,300 | 1,300 | 1,300 |

Note: Sample restricted to caregivers (n=1,300); the dependent variable indicates use of AI chatbots in general. Heteroskedasticity-robust standard errors in parentheses. *** p<0.01, ** p<0.05, * p<0.1.
