# Supplementary material for: Prevalence and predictors of artificial intelligence chatbots use for caregiving: Evidence from the Understanding America Study
Source: PLoS One. 2026 Sep 23;21(9):e0357753. doi: 10.1371/journal.pone.0357753 (PMC13600484; doi:10.1371/journal.pone.0357753)
Supplement: Supplementary file 3 [file pone.0357753.s004.docx]

**July 11, 2026**

**Dear Dr. Ding,**

**Thank you for another opportunity to revise and resubmit our manuscript entitled “Prevalence and Predictors of Artificial Intelligence Chatbots Use: Evidence from the Understanding America Study” submitted for publication in PLOS ONE. We carefully considered Reviewer 1’s remaining comments and revised the manuscript accordingly. Below we describe how the paper was modified in response to each comment. Comments are in bold. Responses are in regular font. Modifications in the manuscript are marked with tracked changes.**

**Reviewer #1:**

**The authors have significantly improved the manuscript in this revised version (R1) (pp. 1, 29). The incorporation of the interrater reliability percentages (Table 3) (pp. 17, 38), the diagnostic evaluation of multicollinearity via Variance Inflation Factors (VIF) (pp. 16, 37), and the explicit justification of the self-definition approach for caregiving constructs successfully resolve the primary methodological concerns raised during the initial review (pp. 22, 43). The manuscript presents a robust mixed-methods design and holds high public health and technological relevance (pp. 1, 9, 30). However, minor technical and statistical clarifications are required before final publication to ensure absolute empirical transparency and internal consistency.

Specific Comments and Technical Queries:**

**1. Statistical and Sample Size Consistency: Discrepancy in Subsample Conditioning (\(N\)): In the narrative text (Page 17, Lines 350–353), the authors state that 25% of the general AI chatbot users (\(N = 487\)) reported utilizing the technology for caregiving (pp. 17, 38). Mathematically, \(25\%\) of \(487\) yields \(121.75\) respondents (rounded to \(122\)). However, Figure 2 explicitly references a sample size of \(N = 121\) for the "Type of Usage" conditional distributions (p. 27). Required Action: Please clarify the exact conditioning mechanism or the handling of missing/omitted data that accounts for this difference of one respondent. Ensure that the text, Figure 2, and the descriptive parameters are synchronized (pp. 17, 27, 38).**

We thank the reviewer for flagging this and for the careful check of our reported figures. To clarify: n=121 is the exact number of caregivers who reported using AI chatbots specifically for caregiving purposes, out of n=487 caregivers who reported using AI chatbots in general (121/487 = 24.85%, rounded to 25% in the text and in Figure 2). The 25% is a rounded percentage calculated directly from these two counts; it is not an independently estimated parameter, so back-multiplying the rounded 25% by 487 (25% × 487 = 121.75) does not recover the exact underlying count and is not the correct way to check consistency with Figure 2.

That said, in the interest of full transparency and to prevent this ambiguity for the reader, we have revised the manuscript to report exact counts alongside every percentage, rather than percentages alone.

**2. Table 4 Regression Sample: Table 4 reports an analytical sample of \(N = 487\) observations across all three nested specifications (pp. 19, 40). This implies that the logit models predict the probability of caregiving-related AI use conditional on being an AI user in general (\(N = 487\)) (pp. 18, 39). Required Action: For clarity, please explicitly modify the table footnote or the methodology section to state that the binary logistic framework restricts the estimation domain to general AI chatbot adopters, rather than the full sample of caregivers (\(N = 1300\)) (pp. 12, 18, 33, 39).**

Thanks for pointing out this inconsistency. We have amended the text in two places to make the estimation sample explicit. First, in the Statistical Analysis section, we clarified: "We then estimated a logistic regression model, among the subsample of caregivers who reported general AI chatbot use (n=487), predicting AI chatbot use for caregiving, specifically." Second, in the Results section describing Table 4, the text now reads: "Table 4 presents the results of regressions, estimated on the sample of caregivers who report having used AI chatbots in general (n=487), predicting the likelihood of using AI chatbots specifically for caregiving purposes." As you suggested, we have also added a footnote to Table 4 stating: "Sample restricted to caregivers who reported general AI chatbot use (n=487); the dependent variable indicates use of AI chatbots specifically for caregiving purposes."

Together, these revisions should clarify which sample we use for the estimation of the Logit regressions in Table 4.

**2. Methodological Rigor in Qualitative Analysis: Thematic Saturation and Conceptualization: While the calculation of percent agreement among three raters (Table 3) is highly commendable and demonstrates strong consensus, the initial generation of the thematic framework was executed by a single investigator (JM) (pp. 17, 38). This introduces a potential vulnerability to confirmation bias during the deductive phase of the exploratory thematic analysis (pp. 22, 43). Required Action: The authors should expand their discussion of limitations (Page 22, Lines 463–466) (pp. 22, 43). Please formally recommend that subsequent implementations utilize independent double-blind thematic coding from inception (rather than just post-hoc verification) to safeguard psychometric validity and inductive objectivity.**

We added on line 465-467, “We recommend that subsequent studies utilize independent double-blind thematic coding from inception, instead of post-hoc verification, to ensure psychometric validity and inductive objectivity.”

**3. Econometric Specification and Reporting: Standard Errors and Clustered Variance: The authors state that the UAS is a probability-based Internet panel representative of the U.S. caregiver population (pp. 11, 32). Given the stratified or complex sampling design characteristic of the UAS architecture, standard ordinary least squares or unweighted logit estimations may yield biased standard errors due to heteroscedasticity or spatial/demographic clustering. Required Action: Please clarify whether the standard errors reported in parentheses in Table 4 are robust standard errors or if they have been adjusted/clustered to account for the complex survey design matrix and sampling weights of the UAS (pp. 19, 40). If sampling weights were applied to compute the marginal effects, please specify this in the Statistical Analysis subsection (pp. 16, 37).**

Regarding sampling weights, we did not apply them in our regression analysis. Our goal in Table 4 is to estimate conditional associations between individual characteristics, caregiving contextual factors, and the likelihood of AI chatbot use for caregiving, rather than to produce population-representative descriptive statistics; for this purpose, unweighted regression is standard practice, since weighting corrects for unequal probabilities of sample selection when estimating population totals or means, but is not generally necessary when the object of interest is a conditional relationship (Solon, Haider, & Wooldridge, 2015).

Second, and more importantly from a practical standpoint, we do not have reliable external benchmarks for the population of U.S. caregivers that would allow us to construct valid sampling weights for our analytic sample. The UAS base panel weights are constructed to render the panel representative of the general U.S. adult population; they are not designed to, and would not correctly, adjust for selection into being a caregiver, nor for the further selection into being an AI chatbot user among caregivers (n=487, the sample underlying Table 4). Applying general population weights to this doubly-selected subsample would not yield valid population-representative estimates for this group, and could introduce additional bias rather than correct for it.

In light of the latter comment, we revisited our language describing the sample throughout the manuscript. While the UAS panel is a probability-based panel benchmarked to the general U.S. adult population, caregiver status is ascertained through a screening question rather than through panel stratification, and no external benchmarks exist for the demographic composition of U.S. caregivers specifically. We therefore cannot verify that our analytic sample is statistically representative of the caregiver population in the technical sense implied by the reviewer's comment. We have revised the text to describe our sample as demographically diverse and drawn from a probability-based panel, rather than as representative of the caregiver population, to avoid overstating this property.

On standard errors, we use heteroskedasticity-robust standard errors in Table 4. We have added this detail both to the Table 4 footnote and to the Statistical Analysis subsection.

**4. Reference Metadata Standardization: Digital Citation Uniformity: Online datasets and web-based publications (e.g., Reference 10: Sidoti & Pew Research, Reference 11: Angrisani et al.) lack full retrieval parameters (p. 24). Required Action: In compliance with PLOS ONE formatting guidelines, please append the exact retrieval dates ([Accessed Day, Month, Year]) and complete URLs where applicable to ensure permanent traceability.**

The following citations have been revised to the required formatting specifications:

10. Sidoti OM, C. 34% of U.S. adults have used ChatGPT, about double the share in 2023. 2025. [Accessed 11, July, 2026] Available: https://www.pewresearch.org/short-reads/2025/06/25/34-of-us-adults-have-used-chatgpt-about-double-the-share-in-2023/

11. Angrisani M, Casanova M, Fast NJ, Narang J, and SchroederJ. Gaps in LLM Awareness, Usage, and Perceptions in the United States: Evidence from a Nationally Representative Longitudinal Survey. PNAS Nexus, Volume 5, Issue 2, February 2026, pgag007, <https://doi.org/10.1093/pnasnexus/pgag007>.

Sincerely,

Joan Monin

Professor

Social and Behavioral Sciences

Yale School of Public Health
