# Supplementary material for: Prevalence and predictors of artificial intelligence chatbots use for caregiving: Evidence from the Understanding America Study
Source: PLoS One. 2026 Sep 23;21(9):e0357753. doi: 10.1371/journal.pone.0357753 (PMC13600484; doi:10.1371/journal.pone.0357753)
Supplement: Supplementary file 4 [file pone.0357753.s003.docx]

**May 20, 2026**

**Dear Dr. Ding,**

**Thank you for the opportunity to revise and resubmit our manuscript entitled “Caregivers’ Use of Artificial Intelligence Chatbots in the Understanding America Study” now revised to “Prevalence and Predictors of Artificial Intelligence Chatbots Use: Evidence from the Understanding America Study” submitted for publication in PLOS ONE. We carefully considered the reviewers’ insightful comments and suggestions and revised the manuscript accordingly. We believe it has improved as a result. Below we describe how the paper was modified in response to each comment. Comments are in bold. Responses are in regular font. Modifications in the manuscript are marked with tracked changes.**

**Editor:**

**1. Please ensure that your manuscript meets PLOS ONE's style requirements, including those for file naming. The PLOS ONE style templates can be found at**

<https://journals.plos.org/plosone/s/file?id=wjVg/PLOSOne_formatting_sample_main_body.pdf>

**and** 
<https://journals.plos.org/plosone/s/file?id=ba62/PLOSOne_formatting_sample_title_authors_affiliations.pdf>.

We have ensured the manuscript meets the requirements, making edits accordingly.

**2.Thank you for stating the following financial disclosure: 
“KB
5R01AG083097-02
National Institute on Aging
https://www.nia.nih.gov/
No “.**

**Please state what role the funders took in the study.  If the funders had no role, please state: "The funders had no role in study design, data collection and analysis, decision to publish, or preparation of the manuscript."**

**If this statement is not correct you must amend it as needed. 
Please include this amended Role of Funder statement in your cover letter; we will change the online submission form on your behalf.**

We added, “The funders had no role in study design, data collection and analysis, decision to publish, or preparation of the manuscript."

**3. We notice that your supplementary tables are included in the manuscript file. Please remove them and upload them with the file type 'Supporting Information'. Please ensure that each Supporting Information file has a legend listed in the manuscript after the references list.**

We removed the table and uploaded the table with the file type S1 Table. We ensured that the Supporting Information file has a legend listed in the manuscript after the references list.

**4. If the reviewer comments include a recommendation to cite specific previously published works, please review and evaluate these publications to determine whether they are relevant and should be cited. There is no requirement to cite these works unless the editor has indicated otherwise.** 

Not applicable.

**Reviewer #1:**

**1. This manuscript examines caregivers’ awareness and use of artificial intelligence chatbots, with a specific focus on caregiving-related use, drawing on data from the Understanding America Study. Overall, the study addresses a timely and relevant topic, and it makes a valuable contribution by leveraging a large, population-representative dataset rather than convenience or pilot samples that dominate much of the existing literature. The manuscript is generally well written, clearly structured, and aligned with the methodological standards e**m**phasized by PLOS ONE. The analyses are appropriate for the research questions posed, and the conclusions are largely supported by the reported results. That said, there are several areas where clarification and additional discussion would strengthen the manuscript and improve the transparency and interpretability of the findings.** **From a methodological standpoint, the study design is sound. The use of a probability-based national panel and clearly defined analytic samples supports the validity of the findings. The description of the participants, measures, and statistical analyses is detailed and mostly clear, allowing for reproducibility. The inclusion of personality traits, cognitive ability, and internet literacy represents a meaningful extension of prior research and is well justified in the introduction. Ethical approval, informed consent procedures, and data availability are clearly documented and meet journal requirements.**

Thanks to the reviewer for your positive comment on this work.

**2. A key issue that would benefit from further clarification concerns the conceptualization and operationalization of “use of AI chatbots for caregiving.” Although respondents were directly asked whether they used chatbots for caregiving purposes, the category itself remains somewhat broad. In particular, both the closed-ended “other” category and several open-ended responses suggest that some reported uses are only indirectly related or not related to caregiving at all, such as work tasks or general information seeking. While this does not invalidate the analyses, it does introduce ambiguity regarding what qualifies as caregiving use. The manuscript would be strengthened by explicitly acknowledging this limitation and clarifying that caregiving use is based on caregivers’ self-definition rather than a narrowly defined functional criterion.**

On page 14, we added the following statement:

“A second potential limitation concerns how we operationalized caregivers’ use of AI chatbots for caregiving purposes. The question eliciting this key measure intentionally relied on respondents’ own judgment of what counts as caregiving-related use, rather than imposing a narrow functional criterion. The examination of open-ended responses indicates that a subset of respondents who reported using chatbots for caregiving purposes described uses that were only indirectly related to caregiving, or, in some cases, unrelated to it (e.g., assistance with work tasks, creative purposes, or general information-seeking). We adopted this self-definition approach deliberately: caregiving research commonly relies on respondents’ self-identification of their caregiving role and responsibilities. In an early descriptive study of an emerging behavior, a narrower functional definition risked excluding novel or context-dependent uses that caregivers themselves consider relevant to their role. We acknowledge, however, that this choice introduces some ambiguity. Our prevalence estimates of caregiving-related AI chatbot use should therefore be interpreted as reflecting caregivers’ own judgments about what qualifies as caregiving use, rather than a narrowly defined functional criterion imposed by the researchers.”

**3. The interpretation of the associations between individual characteristics and chatbot use also warrants greater caution in the discussion. The finding that caregivers with lower cognitive ability and lower internet literacy are more likely to use AI chatbots for caregiving is intriguing but potentially counterintuitive. The current discussion suggests that chatbots may function as an accessible informational resource, but alternative explanations are equally plausible, including greater vulnerability to misinformation or substitution for other sources of support. Given the cross-sectional design, causal interpretations are not possible, and this should be stated more explicitly. A more balanced discussion of possible mechanisms would align better with the evidence presented.**

We note that the original manuscript describes the relationships between individual characteristics and AI chatbot use exclusively in associational terms (e.g., "consistent with the idea that…", "associated with") and does not make any explicit causal claim. Nevertheless, we agree that by proposing the single interpretation of chatbots as an accessible informational resource, we may have implied a stronger interpretive commitment than our data support. We have revised the discussion of this finding on page 13 to consider alternative explanations:

“In addition, we found that those with lower cognitive abilities and internet literacy were more likely to use AI chatbots for caregiving, a pattern that we interpret with caution, given the cross-sectional and observational nature of our data. One plausible mechanism, consistent with open-ended responses describing chatbots as "quick and easy" to use, is that AI chatbots provide an accessible information channel for caregivers who may find it harder to navigate other sources (e.g., medical literature, healthcare professionals, or community-based programs). However, several alternative mechanisms are equally compatible with the observed associations. First, caregivers with lower cognitive ability or internet literacy may be less able to identify the limitations of chatbot output, leaving them more reliant on chatbots without seeking corroboration; conversely, more digitally literate caregivers may be more aware of these risks and deliberately avoid chatbots for caregiving-related decisions. Second, chatbots may substitute for other forms of support, such as professional consultation or peer communities, that less digitally literate caregivers find harder or more costly to access. Third, the observed pattern may partly reflect unobserved characteristics that jointly shape cognitive functioning, internet literacy, and the propensity to engage with novel tools. We could not distinguish between these mechanisms with the data at our disposal. We also note that the identification of the most plausible mechanism(s) is unlikely to be achievable through additional waves of survey data alone, since cognitive ability and internet literacy are largely stable individual characteristics that change slowly, if at all, over the short and medium term.”

We have also added the following paragraph to the section about future research directions:

“A further development is to disentangle the mechanisms underlying the observed negative associations between AI chatbot use for caregiving and both cognitive ability and internet literacy. To this end, future data collection should include richer measures of the intermediate constructs that plausibly link these individual traits to caregiving behavior, such as caregivers’ perceived accuracy of chatbot output, their trust in alternative information sources, and their awareness of chatbot limitations.”

**4. Regarding the qualitative component, the inclusion of open-ended responses is a strength and adds useful context to the quantitative findings. However, the qualitative analysis is necessarily limited by the brevity of responses and the coding process. The initial development of coding themes by a single coder and the use of percent agreement as the reliability measure should be explicitly framed as appropriate for exploratory analysis. While the reported agreement levels are high, a short methodological reflection on the limits of depth, nuance, and interpretive inference in this qualitative component would improve transparency.**

On page 14, we added: “That said, the qualitative analysis was affected by the brevity of responses, and the coding process was limited by the bounds of using this data source. The initial development of coding themes by a single coder and the use of percent agreement as the reliability measure was appropriate for exploratory analysis, but further work is needed to take more rigorous approaches to identifying themes either using inductive approaches or top-down approaches guided by theory. Although rater agreement was high, biases may be present from the one initial coder generating the themes.”

**5. In the statistical analysis, the multivariable models include a large number of covariates that are conceptually related, particularly among the caregiving burden measures and digital skill variables. Although this approach is defensible, the manuscript does not report whether potential collinearity was assessed or considered. A brief statement indicating that collinearity diagnostics were examined, or providing a conceptual justification for including these predictors simultaneously, would strengthen confidence in the reported estimates.**

We have addressed this comment in the revised manuscript.

First, we conducted formal collinearity diagnostics. We computed variance inflation factors (VIFs) for all covariates in the most saturated specification (column 3 of Table 4). All VIFs were well below the conventional threshold of 5: the maximum VIF in the model was 3.07, the mean VIF was 1.83. The VIFs on the variables specifically flagged by the reviewer (caregiving burden and skill measures) were modest (1.81–2.90 for the four burden items; 1.91 for cognitive ability; 1.47 for internet literacy). Pairwise correlations among the four caregiving burden items ranged from 0.41 to 0.74 (the strongest correlation, between "lack of time" and "stress balancing caregiving with other responsibilities," is intuitive given the conceptual proximity of these two items). The correlation between cognitive ability and internet literacy was 0.41, which is moderate and consistent with these variables capturing related but distinct constructs. We have added the following text to the Statistical Analysis section:

“To verify that collinearity does not threaten the stability of our estimates, we computed variance inflation factors (VIFs) for all covariates in the most saturated specification (column 3 of Table 4). The maximum VIF was 3.07, and the mean VIF was 1.83, both well below the conventional threshold of 5.”

Second, we provide a brief conceptual justification for the simultaneous inclusion of these variables. The four burden items, although correlated, tap theoretically distinct dimensions of caregiving burden: temporal pressure (lack of time), role-conflict stress (balancing caregiving with other responsibilities), relational strain (feeling strained when around the care recipient), and role-efficacy uncertainty (uncertainty about how to perform the caregiving role). Similarly, cognitive ability captures general intellectual functioning, whereas internet literacy specifically captures digital skill, a domain more proximate to engagement with a chatbot interface than general cognition. We believe that retaining these variables separately is substantively informative. We added a brief paragraph about this point in the Statistical Analysis section on page 9.

Finally, we note that the coefficients on the variables flagged by the reviewer remain stable in sign, magnitude, and statistical significance across the three nested specifications in Table 4, which sequentially add individual traits and caregiving-related variables to the baseline demographic model. This invariance, alongside the VIF diagnostics, indicates that collinearity is not materially affecting our estimates.

**6. Minor issues include small typographical errors, occasional formatting inconsistencies, and minor transcription errors in quoted open-ended responses. These do not detract from the substance of the work but should be corrected in a revised version.**

We carefully edited the paper throughout.

**7. Additionally, the figures would benefit from clearer labeling of sample sizes and percentages to aid interpretation for readers.**

We have revised Figures 1 and 2 to make sample sizes and percentages more transparent. Each panel now carries a subtitle that explicitly identifies the analytic subsample and its size: "Sample: all caregivers, N=1300" for Figure 1; "Sample: caregivers who reported using a chatbot, N=487" for the top panel of Figure 2; and "Sample: caregivers who reported using a chatbot for caregiving, N=121" for the lower panel of Figure 2. The values reported in each bar are percentages, as indicated by the x-axis label ("percent"), and the exact percentage is shown as a data label next to each bar to aid interpretation. We believe these changes address the reviewer’s comment, but we can further edit the figures if necessary.

**8. In summary, this is a solid and well-executed study that meets the core criteria for publication in PLOS ONE. The manuscript would benefit from revisions that clarify conceptual boundaries, temper interpretative claims, and more explicitly acknowledge methodological limitations, particularly regarding causal inference and qualitative analysis. I recommend revision prior to acceptance, with these points addressed.**

Thank you for helping us improve this paper.

**Reviewer #2:**

**Thank you in advance for conducting this study, which focuses on the contemporary issue of examining caregivers’ awareness and general use of AI chatbots, their use of AI chatbots for caregiving purposes, and variations in these outcomes by individual characteristics (e.g., the Big 5 personality traits, internet literacy, and cognitive ability) and caregiving contextual factors (e.g., caregiving relationship, caregiving stress, and the health condition of the care recipient).**

Thank you for the positive comments on this paper.

**I would like to suggest the following comments.**

**1. In case of a title, a dependent variable of the study could be stated.**

We changed the title to “Prevalence and Predictors of Artificial Intelligence Chatbots Use for Caregiving: Evidence from the Understanding America Study.”

The revised title makes explicit (i) the study’s central dependent variable, namely individuals’ use of AI chatbots for caregiving purposes, and (ii) the study’s two main contributions, namely documenting the prevalence of this behavior and identifying its individual and contextual predictors.

**2. There is confusion between the related term "dependent variable AI awareness" and the independent variable "internet literacy."**

We interpret this comment as raising the possibility that our internet-literacy independent variable and our AI-chatbot awareness/use outcome variables may be capturing overlapping constructs. We appreciate the opportunity to clarify the distinction.

Our internet-literacy measure is operationalized using a 17-item index, which sums dichotomous indicators of familiarity with basic, general-purpose internet concepts and tasks (e.g., navigating browsers, sending email, downloading files, evaluating web addresses; the full item list is documented at https://uasdata.usc.edu/page/Comprehensive+File+And+Panel+Dataset). Crucially, none of the 17 items reference AI tools, chatbots, large language models, or any related technology. Thus, Internet literacy in our analysis captures broad, general-purpose digital skills, while our outcome variables capture awareness and use of a specific, recently emerged class of technology (AI chatbots such as ChatGPT, Gemini, Copilot, etc.). The two constructs are conceptually distinct, and the empirical relationship between them is itself a substantive object of inquiry in our paper rather than a definitional overlap.

To make this distinction explicit for the reader, we have revised the description of the internet-literacy measure in the Methods section to clarify that the underlying items concern general internet skills only and do not include AI-specific content. The revised passage reads:

**3. Other researchers like Walter Mischel (2004) and Angrisani M., et al. (2025) showed that AI chatbot usage is associated with personality traits, especially with openness, so your rationale for conducting this research is not clear other than repetition.**

The Mischel (2004) reference is in support of our rationale for our study to focus on both personality and caregiving contextual factors because of Walter Mischel’s work showing that people both have enduring qualities that can be captured by personality, but they also respond to situations, which suggests measuring contextual factors. The Angrisani, et al (2025) study showed that personality traits was related to AI awareness and usage in general, but not related to caregiving. We clarify on page 4 that “No prior study has examined personality traits related to AI use for caregiving purposes.”

**4. Some irrelevant sentence or lack of clarity on page 4 "Within the UAS, the authors of this paper lead a project called UAS-Caregiving Life course Experiences Assessed in Real-time (UAS-CLEAR)."**

We revised the sentence on page 4 to “Within the UAS, the authors of this paper lead a project called the “UAS-Caregiving Lifecourse Experiences Assessed in Real-time (UAS-CLEAR)” Study.”

The sentence that follows clarifies that the UAS-CLEAR study is a UAS project focused on understanding caregivers’ daily experiences and well-being through longitudinal surveys.

**5. The way you check reliability and validity of measurement tools for cognitive ability, internet literacy, and personality tests should be described.**

We have addressed both the reliability and validity of our measurement tools as follows.

*Reliability.* We computed Cronbach's alpha for each multi-item measure in our analytic sample (N = 1,300). All measures exhibit acceptable to excellent internal consistency, with values at or above the conventional threshold of 0.70 used in survey research:

Personality traits:

Neuroticism, α = 0.84; Extroversion, α = 0.82; Openness, α = 0.78; Agreeableness, α = 0.77; Conscientiousness, α = 0.81

Internet literacy, α = 0.90

Cognitive ability, α = 0.77

These reliability statistics have been added to the Methods section as footnotes attached to the descriptions of the corresponding measures.

*Validity*. The three measurement instruments in question are widely used in social-science research and have undergone extensive validation in prior work, which we reference explicitly in the Methods section.

Big-5 personality traits. The Big-5 items used by the UAS are derived from the standard inventory developed by McCrae and Costa (1999) and elaborated in McCrae (2004), which is one of the most extensively validated personality instruments in the literature and has been administered in dozens of nationally representative surveys worldwide.

Cognitive ability. Cognitive ability is measured using three subtests of the Woodcock-Johnson IV Tests of Cognitive Abilities (Mather & Jaffe, 2016), a clinically standardized instrument with population norms (T-scores: mean = 50, SD = 10) and a long-established record of construct, criterion, and content validity. The mean composite score in our sample (152) is consistent with population norms.

Internet literacy. Internet literacy is measured using the UAS panel's 17-item index, which has been administered to the UAS panel across multiple waves and is documented in detail by the UAS team (https://uasdata.usc.edu/page/Comprehensive+File+And+Panel+Dataset). Its content captures basic, general-purpose internet skills (browsing, email, file management), a construct closely aligned with established digital-skills inventories.

**6. The research design you employed is not clearly stated in both the method and abstract sections of your study.**

We agree that the research design was not stated explicitly in either the abstract or the Methods section. We have addressed this in both locations.

In the abstract, we have added a sentence specifying that the study employs a cross-sectional, mixed-methods design using a single wave of the UAS-CLEAR panel.

In the Methods section, we have added a labeled “Research Design” sub-section, clarifying that the analysis is cross-sectional, observational, and mixed-methods.

We hope these additions provide a clearer and more conventional statement of the study’s research design.

Sincerely,

Joan Monin

Professor

Social and Behavioral Sciences

Yale School of Public Health
