# Supplementary material for: Prevalence and predictors of artificial intelligence chatbots use for caregiving: Evidence from the Understanding America Study
Source: PLoS One. 2026 Sep 23;21(9):e0357753. doi: 10.1371/journal.pone.0357753 (PMC13600484; doi:10.1371/journal.pone.0357753)
Supplement: Peer review history

# Peer review: Decision Letter 0

**DOI:** 10.1371/journal.pone.0357753.r001

**Authors:** Yi Ding

23 Apr 2026

Dear Dr. Monin,

Thank you for submitting your manuscript to PLOS ONE. After careful consideration, we feel that it has merit but does not fully meet PLOS ONE’s publication criteria as it currently stands. Therefore, we invite you to submit a revised version of the manuscript that addresses the points raised during the review process.

Please submit your revised manuscript by Jun 07 2026 11:59PM. If you will need more time than this to complete your revisions, please reply to this message or contact the journal office at plosone@plos.org. When you're ready to submit your revision, log on to https://www.editorialmanager.com/pone/ and select the 'Submissions Needing Revision' folder to locate your manuscript file.

- A letter that responds to each point raised by the academic editor and reviewer(s). You should upload this letter as a separate file labeled 'Response to Reviewers'
- A marked-up copy of your manuscript that highlights changes made to the original version. You should upload this as a separate file labeled 'Revised Manuscript with Track Changes'.
- An unmarked version of your revised paper without tracked changes. You should upload this as a separate file labeled 'Manuscript'.

If you would like to make changes to your financial disclosure, please include your updated statement in your cover letter. Guidelines for resubmitting your figure files are available below the reviewer comments at the end of this letter.

If applicable, we recommend that you deposit your laboratory protocols in protocols.io to enhance the reproducibility of your results. Protocols.io assigns your protocol its own identifier (DOI) so that it can be cited independently in the future. For instructions see: https://journals.plos.org/plosone/s/submission-guidelines#loc-laboratory-protocols. Additionally, PLOS ONE offers an option for publishing peer-reviewed Lab Protocol articles, which describe protocols hosted on protocols.io. Read more information on sharing protocols at https://plos.org/protocols?utm_medium=editorial-email&utm_source=authorletters&utm_campaign=protocols.

As the corresponding author, your ORCID iD is verified in the submission system and will appear in the published article. PLOS supports the use of ORCID, and we encourage all coauthors to register for an ORCID iD and use it as well. Please encourage your coauthors to verify their ORCID iD within the submission system before final acceptance, as unverified ORCID iDs will not appear in the published article. *Only*  the individual author can complete the verification step; PLOS staff *cannot*  verify ORCID iDs on behalf of authors.

We look forward to receiving your revised manuscript.

Kind regards,

Yi Ding

Academic Editor

PLOS One

Journal Requirements:

When submitting your revision, we need you to address these additional requirements.

1. Please ensure that your manuscript meets PLOS ONE's style requirements, including those for file naming. The PLOS ONE style templates can be found at

https://journals.plos.org/plosone/s/file?id=wjVg/PLOSOne_formatting_sample_main_body.pdf and

https://journals.plos.org/plosone/s/file?id=ba62/PLOSOne_formatting_sample_title_authors_affiliations.pdf.

2.Thank you for stating the following financial disclosure:

“KB

5R01AG083097-02

National Institute on Aging

https://www.nia.nih.gov/

No “.

Please state what role the funders took in the study.  If the funders had no role, please state: "The funders had no role in study design, data collection and analysis, decision to publish, or preparation of the manuscript."

If this statement is not correct you must amend it as needed.

Please include this amended Role of Funder statement in your cover letter; we will change the online submission form on your behalf.

3. We notice that your supplementary tables are included in the manuscript file. Please remove them and upload them with the file type 'Supporting Information'. Please ensure that each Supporting Information file has a legend listed in the manuscript after the references list.

4. If the reviewer comments include a recommendation to cite specific previously published works, please review and evaluate these publications to determine whether they are relevant and should be cited. There is no requirement to cite these works unless the editor has indicated otherwise.

[Note: HTML markup is below. Please do not edit.]

Reviewers' comments:

Reviewer's Responses to Questions

**Comments to the Author**

1. Is the manuscript technically sound, and do the data support the conclusions?

Reviewer #1: Yes

Reviewer #2: Yes

**********

2. Has the statistical analysis been performed appropriately and rigorously?

Reviewer #1: Yes

Reviewer #2: Yes

**********

3. Have the authors made all data underlying the findings in their manuscript fully available?

The PLOS Data policy

Reviewer #1: Yes

Reviewer #2: Yes

**********

4. Is the manuscript presented in an intelligible fashion and written in standard English?

Reviewer #1: Yes

Reviewer #2: No

**********

Reviewer #1: Reviewer Report

This manuscript examines caregivers’ awareness and use of artificial intelligence chatbots, with a specific focus on caregiving-related use, drawing on data from the Understanding America Study. Overall, the study addresses a timely and relevant topic, and it makes a valuable contribution by leveraging a large, population-representative dataset rather than convenience or pilot samples that dominate much of the existing literature. The manuscript is generally well written, clearly structured, and aligned with the methodological standards emphasized by PLOS ONE. The analyses are appropriate for the research questions posed, and the conclusions are largely supported by the reported results. That said, there are several areas where clarification and additional discussion would strengthen the manuscript and improve the transparency and interpretability of the findings.

From a methodological standpoint, the study design is sound. The use of a probability-based national panel and clearly defined analytic samples supports the validity of the findings. The description of the participants, measures, and statistical analyses is detailed and mostly clear, allowing for reproducibility. The inclusion of personality traits, cognitive ability, and internet literacy represents a meaningful extension of prior research and is well justified in the introduction. Ethical approval, informed consent procedures, and data availability are clearly documented and meet journal requirements.

A key issue that would benefit from further clarification concerns the conceptualization and operationalization of “use of AI chatbots for caregiving.” Although respondents were directly asked whether they used chatbots for caregiving purposes, the category itself remains somewhat broad. In particular, both the closed-ended “other” category and several open-ended responses suggest that some reported uses are only indirectly related or not related to caregiving at all, such as work tasks or general information seeking. While this does not invalidate the analyses, it does introduce ambiguity regarding what qualifies as caregiving use. The manuscript would be strengthened by explicitly acknowledging this limitation and clarifying that caregiving use is based on caregivers’ self-definition rather than a narrowly defined functional criterion.

The interpretation of the associations between individual characteristics and chatbot use also warrants greater caution in the discussion. The finding that caregivers with lower cognitive ability and lower internet literacy are more likely to use AI chatbots for caregiving is intriguing but potentially counterintuitive. The current discussion suggests that chatbots may function as an accessible informational resource, but alternative explanations are equally plausible, including greater vulnerability to misinformation or substitution for other sources of support. Given the cross-sectional design, causal interpretations are not possible, and this should be stated more explicitly. A more balanced discussion of possible mechanisms would align better with the evidence presented.

Regarding the qualitative component, the inclusion of open-ended responses is a strength and adds useful context to the quantitative findings. However, the qualitative analysis is necessarily limited by the brevity of responses and the coding process. The initial development of coding themes by a single coder and the use of percent agreement as the reliability measure should be explicitly framed as appropriate for exploratory analysis. While the reported agreement levels are high, a short methodological reflection on the limits of depth, nuance, and interpretive inference in this qualitative component would improve transparency.

In the statistical analysis, the multivariable models include a large number of covariates that are conceptually related, particularly among the caregiving burden measures and digital skill variables. Although this approach is defensible, the manuscript does not report whether potential collinearity was assessed or considered. A brief statement indicating that collinearity diagnostics were examined, or providing a conceptual justification for including these predictors simultaneously, would strengthen confidence in the reported estimates.

Minor issues include small typographical errors, occasional formatting inconsistencies, and minor transcription errors in quoted open-ended responses. These do not detract from the substance of the work but should be corrected in a revised version. Additionally, the figures would benefit from clearer labeling of sample sizes and percentages to aid interpretation for readers.

In summary, this is a solid and well-executed study that meets the core criteria for publication in PLOS ONE. The manuscript would benefit from revisions that clarify conceptual boundaries, temper interpretative claims, and more explicitly acknowledge methodological limitations, particularly regarding causal inference and qualitative analysis. I recommend revision prior to acceptance, with these points addressed.

Reviewer #2: Thank you in advance for conducting this study, which focuses on the contemporary issue of examining caregivers’ awareness and general use of AI chatbots, their use of AI chatbots for caregiving purposes, and variations in these outcomes by individual characteristics (e.g., the Big 5 personality traits, internet literacy, and cognitive ability) and caregiving contextual factors (e.g., caregiving relationship, caregiving stress, and the health condition of the care recipient).

I would like to suggest the following comments.

1. In case of a title, a dependent variable of the study could be stated.

2. There is confusion between the related term "dependent variable AI awareness" and the independent variable "internet literacy."

3. Other researchers like Walter Mischel (2004) and Angrisani M., et al. (2025) showed that AI chatbot usage is associated with personality traits, especially with openness, so your rationale for conducting this research is not clear other than repetition.

4. Some irrelevant sentence or lack of clarity on page 4 "Within the UAS, the authors of this paper lead a project called UAS-Caregiving Life course Experiences Assessed in Real-time (UAS-CLEAR)."

5. The way you check reliability and validity of measurement tools for cognitive ability, internet literacy, and personality tests should be described.

6. The research design you employed is not clearly stated in both the method and abstract sections of your study.

**********

what does this mean?). If published, this will include your full peer review and any attached files.

If you choose “no”, your identity will remain anonymous but your review may still be made public.

**Do you want your identity to be public for this peer review?**  For information about this choice, including consent withdrawal, please see our Privacy Policy

Reviewer #1: No

Reviewer #2: No

**********

[NOTE: If reviewer comments were submitted as an attachment file, they will be attached to this email and accessible via the submission site. Please log into your account, locate the manuscript record, and check for the action link "View Attachments". If this link does not appear, there are no attachment files.]

To ensure your figures meet our technical requirements, please review our figure guidelines: https://journals.plos.org/plosone/s/figures

You may also use PLOS’s free figure tool, NAAS, to help you prepare publication quality figures: https://journals.plos.org/plosone/s/figures#loc-tools-for-figure-preparation.

NAAS will assess whether your figures meet our technical requirements by comparing each figure against our figure specifications.

# Author comment: Author response to Decision Letter 1

**DOI:** 10.1371/journal.pone.0357753.r002

22 May 2026

See attached file "Response to Reviewers".

Attachment Submitted filename: Response to reviewers 5 22 26 chat bot.docx

# Peer review: Decision Letter 1

**DOI:** 10.1371/journal.pone.0357753.r003

**Authors:** Yi Ding

5 Jul 2026

Dear Dr. Monin,

Thank you for submitting your manuscript to PLOS ONE. After careful consideration, we feel that it has merit but does not fully meet PLOS ONE’s publication criteria as it currently stands. Therefore, we invite you to submit a revised version of the manuscript that addresses the points raised during the review process.

Please submit your revised manuscript by Aug 19 2026 11:59PM. If you will need more time than this to complete your revisions, please reply to this message or contact the journal office at plosone@plos.org. When you're ready to submit your revision, log on to https://www.editorialmanager.com/pone/ and select the 'Submissions Needing Revision' folder to locate your manuscript file.

- A letter that responds to each point raised by the academic editor and reviewer(s). You should upload this letter as a separate file labeled 'Response to Reviewers'.
- A marked-up copy of your manuscript that highlights changes made to the original version. You should upload this as a separate file labeled 'Revised Manuscript with Track Changes'.
- An unmarked version of your revised paper without tracked changes. You should upload this as a separate file labeled 'Manuscript'.

If you would like to make changes to your financial disclosure, please include your updated statement in your cover letter. Guidelines for resubmitting your figure files are available below the reviewer comments at the end of this letter.

If applicable, we recommend that you deposit your laboratory protocols in protocols.io to enhance the reproducibility of your results. Protocols.io assigns your protocol its own identifier (DOI) so that it can be cited independently in the future. For instructions see: https://journals.plos.org/plosone/s/submission-guidelines#loc-laboratory-protocols. Additionally, PLOS ONE offers an option for publishing peer-reviewed Lab Protocol articles, which describe protocols hosted on protocols.io. Read more information on sharing protocols at https://plos.org/protocols?utm_medium=editorial-email&utm_source=authorletters&utm_campaign=protocols.

As the corresponding author, your ORCID iD is verified in the submission system and will appear in the published article. PLOS supports the use of ORCID, and we encourage all coauthors to register for an ORCID iD and use it as well. Please encourage your coauthors to verify their ORCID iD within the submission system before final acceptance, as unverified ORCID iDs will not appear in the published article. *Only*  the individual author can complete the verification step; PLOS staff *cannot*  verify ORCID iDs on behalf of authors.

We look forward to receiving your revised manuscript.

Kind regards,

Yi Ding

Academic Editor

PLOS One

**Journal Requirements:**

1. If the reviewer comments include a recommendation to cite specific previously published works, please review and evaluate these publications to determine whether they are relevant and should be cited. There is no requirement to cite these works unless the editor has indicated otherwise.

2. Please review your reference list to ensure that it is complete and correct. If you have cited papers that have been retracted, please include the rationale for doing so in the manuscript text, or remove these references and replace them with relevant current references. Any changes to the reference list should be mentioned in the rebuttal letter that accompanies your revised manuscript. If you need to cite a retracted article, indicate the article’s retracted status in the References list and also include a citation and full reference for the retraction notice.

[Note: HTML markup is below. Please do not edit.]

Reviewers' comments:

Reviewer's Responses to Questions

**Comments to the Author**

Reviewer #1: All comments have been addressed

Reviewer #2: All comments have been addressed

**********

2. Is the manuscript technically sound, and do the data support the conclusions?

Reviewer #1: Yes

Reviewer #2: Yes

**********

3. Has the statistical analysis been performed appropriately and rigorously?

Reviewer #1: Yes

Reviewer #2: Yes

**********

4. Have the authors made all data underlying the findings in their manuscript fully available?

The PLOS Data policy

Reviewer #1: Yes

Reviewer #2: Yes

**********

5. Is the manuscript presented in an intelligible fashion and written in standard English?

Reviewer #1: Yes

Reviewer #2: Yes

**********

**Reviewer #1:** Manuscript Number: PONE-D-26-08410R1

Title: Prevalence and Predictors of Artificial Intelligence Chatbots Use for Caregiving: Evidence from the Understanding America Study

Recommendation: Minor Revision

General Comments

The authors have significantly improved the manuscript in this revised version (R1) (pp. 1, 29). The incorporation of the interrater reliability percentages (Table 3) (pp. 17, 38), the diagnostic evaluation of multicollinearity via Variance Inflation Factors (VIF) (pp. 16, 37), and the explicit justification of the self-definition approach for caregiving constructs successfully resolve the primary methodological concerns raised during the initial review (pp. 22, 43). The manuscript presents a robust mixed-methods design and holds high public health and technological relevance (pp. 1, 9, 30). However, minor technical and statistical clarifications are required before final publication to ensure absolute empirical transparency and internal consistency.

________________________________________

Specific Comments and Technical Queries

1. Statistical and Sample Size Consistency

• Discrepancy in Subsample Conditioning (\(N\)): In the narrative text (Page 17, Lines 350–353), the authors state that 25% of the general AI chatbot users (\(N = 487\)) reported utilizing the technology for caregiving (pp. 17, 38). Mathematically, \(25\%\) of \(487\) yields \(121.75\) respondents (rounded to \(122\)). However, Figure 2 explicitly references a sample size of \(N = 121\) for the "Type of Usage" conditional distributions (p. 27).

o Required Action: Please clarify the exact conditioning mechanism or the handling of missing/omitted data that accounts for this difference of one respondent. Ensure that the text, Figure 2, and the descriptive parameters are synchronized (pp. 17, 27, 38).

• Table 4 Regression Sample: Table 4 reports an analytical sample of \(N = 487\) observations across all three nested specifications (pp. 19, 40). This implies that the logit models predict the probability of caregiving-related AI use conditional on being an AI user in general (\(N = 487\)) (pp. 18, 39).

o Required Action: For clarity, please explicitly modify the table footnote or the methodology section to state that the binary logistic framework restricts the estimation domain to general AI chatbot adopters, rather than the full sample of caregivers (\(N = 1300\)) (pp. 12, 18, 33, 39).

2. Methodological Rigor in Qualitative Analysis

• Thematic Saturation and Conceptualization: While the calculation of percent agreement among three raters (Table 3) is highly commendable and demonstrates strong consensus, the initial generation of the thematic framework was executed by a single investigator (JM) (pp. 17, 38). This introduces a potential vulnerability to confirmation bias during the deductive phase of the exploratory thematic analysis (pp. 22, 43).

o Required Action: The authors should expand their discussion of limitations (Page 22, Lines 463–466) (pp. 22, 43). Please formally recommend that subsequent implementations utilize independent double-blind thematic coding from inception (rather than just post-hoc verification) to safeguard psychometric validity and inductive objectivity.

3. Econometric Specification and Reporting

• Standard Errors and Clustered Variance: The authors state that the UAS is a probability-based Internet panel representative of the U.S. caregiver population (pp. 11, 32). Given the stratified or complex sampling design characteristic of the UAS architecture, standard ordinary least squares or unweighted logit estimations may yield biased standard errors due to heteroscedasticity or spatial/demographic clustering.

o Required Action: Please clarify whether the standard errors reported in parentheses in Table 4 are robust standard errors or if they have been adjusted/clustered to account for the complex survey design matrix and sampling weights of the UAS (pp. 19, 40). If sampling weights were applied to compute the marginal effects, please specify this in the Statistical Analysis subsection (pp. 16, 37).

4. Reference Metadata Estandardization

• Digital Citation Uniformity: Online datasets and web-based publications (e.g., Reference 10: Sidoti & Pew Research, Reference 11: Angrisani et al.) lack full retrieval parameters (p. 24).

o Required Action: In compliance with PLOS ONE formatting guidelines, please append the exact retrieval dates ([Accessed Day, Month, Year]) and complete URLs where applicable to ensure permanent traceability.

**Reviewer #2:** This manuscript entitled Prevalence and Predictors of Artificial Intelligence Chatbots Use for Caregiving: Evidence from the Understanding America Study, examines caregivers’ awareness and use of artificial intelligence

Chatbots: In addition to addressing a timely and relevant topic, the author of the manuscript takes serious remedial action for my first comments. Generally this manuscript is well written, clearly structured, and aligned with

the methodological standards emphasized by PLOS ONE.

**********

what does this mean?). If published, this will include your full peer review and any attached files.

If you choose “no”, your identity will remain anonymous but your review may still be made public.

**Do you want your identity to be public for this peer review?**  For information about this choice, including consent withdrawal, please see our Privacy Policy

Reviewer #1: No

Reviewer #2: No

**********

[NOTE: If reviewer comments were submitted as an attachment file, they will be attached to this email and accessible via the submission site. Please log into your account, locate the manuscript record, and check for the action link "View Attachments". If this link does not appear, there are no attachment files.]

To ensure your figures meet our technical requirements, please review our figure guidelines: https://journals.plos.org/plosone/s/figures

You may also use PLOS’s free figure tool, NAAS, to help you prepare publication quality figures: https://journals.plos.org/plosone/s/figures#loc-tools-for-figure-preparation.

NAAS will assess whether your figures meet our technical requirements by comparing each figure against our figure specifications.

# Author comment: Author response to Decision Letter 2

**DOI:** 10.1371/journal.pone.0357753.r004

16 Jul 2026

July 11, 2026

Dear Dr. Ding,

Thank you for another opportunity to revise and resubmit our manuscript entitled “Prevalence and Predictors of Artificial Intelligence Chatbots Use: Evidence from the Understanding America Study” submitted for publication in PLOS ONE. We carefully considered Reviewer 1’s remaining comments and revised the manuscript accordingly. Below we describe how the paper was modified in response to each comment. Comments are in bold. Responses are in regular font. Modifications in the manuscript are marked with tracked changes.

Reviewer #1:

The authors have significantly improved the manuscript in this revised version (R1) (pp. 1, 29). The incorporation of the interrater reliability percentages (Table 3) (pp. 17, 38), the diagnostic evaluation of multicollinearity via Variance Inflation Factors (VIF) (pp. 16, 37), and the explicit justification of the self-definition approach for caregiving constructs successfully resolve the primary methodological concerns raised during the initial review (pp. 22, 43). The manuscript presents a robust mixed-methods design and holds high public health and technological relevance (pp. 1, 9, 30). However, minor technical and statistical clarifications are required before final publication to ensure absolute empirical transparency and internal consistency.

Specific Comments and Technical Queries:

1. Statistical and Sample Size Consistency: Discrepancy in Subsample Conditioning (\(N\)): In the narrative text (Page 17, Lines 350–353), the authors state that 25% of the general AI chatbot users (\(N = 487\)) reported utilizing the technology for caregiving (pp. 17, 38). Mathematically, \(25\%\) of \(487\) yields \(121.75\) respondents (rounded to \(122\)). However, Figure 2 explicitly references a sample size of \(N = 121\) for the "Type of Usage" conditional distributions (p. 27). Required Action: Please clarify the exact conditioning mechanism or the handling of missing/omitted data that accounts for this difference of one respondent. Ensure that the text, Figure 2, and the descriptive parameters are synchronized (pp. 17, 27, 38).

We thank the reviewer for flagging this and for the careful check of our reported figures. To clarify: n=121 is the exact number of caregivers who reported using AI chatbots specifically for caregiving purposes, out of n=487 caregivers who reported using AI chatbots in general (121/487 = 24.85%, rounded to 25% in the text and in Figure 2). The 25% is a rounded percentage calculated directly from these two counts; it is not an independently estimated parameter, so back-multiplying the rounded 25% by 487 (25% × 487 = 121.75) does not recover the exact underlying count and is not the correct way to check consistency with Figure 2.

That said, in the interest of full transparency and to prevent this ambiguity for the reader, we have revised the manuscript to report exact counts alongside every percentage, rather than percentages alone.

2. Table 4 Regression Sample: Table 4 reports an analytical sample of \(N = 487\) observations across all three nested specifications (pp. 19, 40). This implies that the logit models predict the probability of caregiving-related AI use conditional on being an AI user in general (\(N = 487\)) (pp. 18, 39). Required Action: For clarity, please explicitly modify the table footnote or the methodology section to state that the binary logistic framework restricts the estimation domain to general AI chatbot adopters, rather than the full sample of caregivers (\(N = 1300\)) (pp. 12, 18, 33, 39).

Thanks for pointing out this inconsistency. We have amended the text in two places to make the estimation sample explicit. First, in the Statistical Analysis section, we clarified: "We then estimated a logistic regression model, among the subsample of caregivers who reported general AI chatbot use (n=487), predicting AI chatbot use for caregiving, specifically." Second, in the Results section describing Table 4, the text now reads: "Table 4 presents the results of regressions, estimated on the sample of caregivers who report having used AI chatbots in general (n=487), predicting the likelihood of using AI chatbots specifically for caregiving purposes." As you suggested, we have also added a footnote to Table 4 stating: "Sample restricted to caregivers who reported general AI chatbot use (n=487); the dependent variable indicates use of AI chatbots specifically for caregiving purposes."

Together, these revisions should clarify which sample we use for the estimation of the Logit regressions in Table 4.

2. Methodological Rigor in Qualitative Analysis: Thematic Saturation and Conceptualization: While the calculation of percent agreement among three raters (Table 3) is highly commendable and demonstrates strong consensus, the initial generation of the thematic framework was executed by a single investigator (JM) (pp. 17, 38). This introduces a potential vulnerability to confirmation bias during the deductive phase of the exploratory thematic analysis (pp. 22, 43). Required Action: The authors should expand their discussion of limitations (Page 22, Lines 463–466) (pp. 22, 43). Please formally recommend that subsequent implementations utilize independent double-blind thematic coding from inception (rather than just post-hoc verification) to safeguard psychometric validity and inductive objectivity.

We added on line 465-467, “We recommend that subsequent studies utilize independent double-blind thematic coding from inception, instead of post-hoc verification, to ensure psychometric validity and inductive objectivity.”

3. Econometric Specification and Reporting: Standard Errors and Clustered Variance: The authors state that the UAS is a probability-based Internet panel representative of the U.S. caregiver population (pp. 11, 32). Given the stratified or complex sampling design characteristic of the UAS architecture, standard ordinary least squares or unweighted logit estimations may yield biased standard errors due to heteroscedasticity or spatial/demographic clustering. Required Action: Please clarify whether the standard errors reported in parentheses in Table 4 are robust standard errors or if they have been adjusted/clustered to account for the complex survey design matrix and sampling weights of the UAS (pp. 19, 40). If sampling weights were applied to compute the marginal effects, please specify this in the Statistical Analysis subsection (pp. 16, 37).

Regarding sampling weights, we did not apply them in our regression analysis. Our goal in Table 4 is to estimate conditional associations between individual characteristics, caregiving contextual factors, and the likelihood of AI chatbot use for caregiving, rather than to produce population-representative descriptive statistics; for this purpose, unweighted regression is standard practice, since weighting corrects for unequal probabilities of sample selection when estimating population totals or means, but is not generally necessary when the object of interest is a conditional relationship (Solon, Haider, & Wooldridge, 2015).

Second, and more importantly from a practical standpoint, we do not have reliable external benchmarks for the population of U.S. caregivers that would allow us to construct valid sampling weights for our analytic sample. The UAS base panel weights are constructed to render the panel representative of the general U.S. adult population; they are not designed to, and would not correctly, adjust for selection into being a caregiver, nor for the further selection into being an AI chatbot user among caregivers (n=487, the sample underlying Table 4). Applying general population weights to this doubly-selected subsample would not yield valid population-representative estimates for this group, and could introduce additional bias rather than correct for it.

In light of the latter comment, we revisited our language describing the sample throughout the manuscript. While the UAS panel is a probability-based panel benchmarked to the general U.S. adult population, caregiver status is ascertained through a screening question rather than through panel stratification, and no external benchmarks exist for the demographic composition of U.S. caregivers specifically. We therefore cannot verify that our analytic sample is statistically representative of the caregiver population in the technical sense implied by the reviewer's comment. We have revised the text to describe our sample as demographically diverse and drawn from a probability-based panel, rather than as representative of the caregiver population, to avoid overstating this property.

On standard errors, we use heteroskedasticity-robust standard errors in Table 4. We have added this detail both to the Table 4 footnote and to the Statistical Analysis subsection.

4. Reference Metadata Standardization: Digital Citation Uniformity: Online datasets and web-based publications (e.g., Reference 10: Sidoti & Pew Research, Reference 11: Angrisani et al.) lack full retrieval parameters (p. 24). Required Action: In compliance with PLOS ONE formatting guidelines, please append the exact retrieval dates ([Accessed Day, Month, Year]) and complete URLs where applicable to ensure permanent traceability.

The following citations have been revised to the required formatting specifications:

10. Sidoti OM, C. 34% of U.S. adults have used ChatGPT, about double the share in 2023. 2025. [Accessed 11, July, 2026] Available: https://www.pewresearch.org/short-reads/2025/06/25/34-of-us-adults-have-used-chatgpt-about-double-the-share-in-2023/

11. Angrisani M, Casanova M, Fast NJ, Narang J, and SchroederJ. Gaps in LLM Awareness, Usage, and Perceptions in the United States: Evidence from a Nationally Representative Longitudinal Survey. PNAS Nexus, Volume 5, Issue 2, February 2026, pgag007, https://doi.org/10.1093/pnasnexus/pgag007.

Sincerely,

Joan Monin

Professor

Social and Behavioral Sciences

Yale School of Public Health

Attachment Submitted filename: Response to reviewers 7 16 2026 chatbotjm.docx

# Peer review: Decision Letter 2

**DOI:** 10.1371/journal.pone.0357753.r005

**Authors:** Yi Ding

23 Aug 2026

Prevalence and Predictors of Artificial Intelligence Chatbots Use for Caregiving: Evidence from the Understanding America Study

PONE-D-26-08410R2

Dear Dr. Monin,

We’re pleased to inform you that your manuscript has been judged scientifically suitable for publication and will be formally accepted for publication once it meets all outstanding technical requirements.

Within one week, you’ll receive an e-mail detailing the required amendments. When these have been addressed, you’ll receive a formal acceptance letter and your manuscript will be scheduled for publication.

An invoice will be generated when your article is formally accepted. Please note, if your institution has a publishing partnership with PLOS and your article meets the relevant criteria, all or part of your publication costs will be covered. Please make sure your user information is up-to-date by logging into Editorial Manager at Editorial Manager® and clicking the ‘Update My Information' link at the top of the page. For questions related to billing, please contact billing support.

If your institution or institutions have a press office, please notify them about your upcoming paper to help maximize its impact. If they’ll be preparing press materials, please inform our press team as soon as possible -- no later than 48 hours after receiving the formal acceptance. Your manuscript will remain under strict press embargo until 2 pm Eastern Time on the date of publication. For more information, please contact onepress@plos.org.

Kind regards,

Yi Ding

Academic Editor

PLOS One

Additional Editor Comments (optional):

Reviewers' comments:

Reviewer's Responses to Questions

**Comments to the Author**

Reviewer #1: All comments have been addressed

Reviewer #2: All comments have been addressed

**********

2. Is the manuscript technically sound, and do the data support the conclusions?

Reviewer #1: Yes

Reviewer #2: Yes

**********

3. Has the statistical analysis been performed appropriately and rigorously?

Reviewer #1: Yes

Reviewer #2: Yes

**********

4. Have the authors made all data underlying the findings in their manuscript fully available?

The PLOS Data policy

Reviewer #1: Yes

Reviewer #2: Yes

**********

5. Is the manuscript presented in an intelligible fashion and written in standard English?

Reviewer #1: Yes

Reviewer #2: Yes

**********

Reviewer #1: PLOS ONE – Manuscript Review Report

Manuscript Number: PONE-D-26-08410R2

Title: Prevalence and Predictors of Artificial Intelligence Chatbots Use for Caregiving: Evidence from the Understanding America Study

Article Type: Research Article

________________________________________

General Evaluation

The manuscript has undergone highly significant methodological and empirical improvements compared to its previous versions. The authors have outstandingly addressed the core concerns raised during the prior two rounds of review. Specifically, the inclusion of the interrater reliability percentages (Table 3), the formal diagnostic evaluation of multicollinearity via Variance Inflation Factors (VIF), and the methodological justification of the self-definition approach for caregiving constructs successfully reinforce the technical robustness of the mixed-methods design.

The study holds high relevance and novelty at the intersection of public health, personality psychology, and emerging technologies, offering one of the first population-based snapshots of how family caregivers interact with generative AI tools.

Below are the final analytical clarifications and minor methodological verifications noted prior to recommending final acceptance for publication.

________________________________________

Specific Comments and Technical Queries

1. Statistical and Sample Size Consistency (\(N\))

• Comment: In the narrative text, it is correctly detailed that 25% of the general AI chatbot users (\(n = 487\)) utilized the technology for caregiving tasks. Mathematically, this would yield \(\approx 122\) respondents. However, Figure 2 explicitly restricts the conditional distribution for "Type of Usage" to a sample size of \(N = 121\).

• Assessment of Revision: The authors’ intellectual honesty and transparency in clarifying that \(121/487 = 24.85\%\) (which was rounded to 25% in the text) is highly appreciated. The decision to report exact underlying counts alongside every percentage in this revised version is excellent, as it removes any calculation ambiguity for the reader.

2. Table 4 Regression Sample and Restriction of Estimation Domain

• Comment: Table 4 reports a stable analytical sample of \(N = 487\) observations across all three nested specifications. This raised questions regarding whether the logit models predicted the probability across the full caregiver sample (\(N = 1300\)) or exclusively among prior AI adopters.

• Assessment of Revision: The modifications introduced in the Statistical Analysis section, the Results section, and particularly the addition of the footnote to Table 4 entirely resolve this concern. It is now methodologically transparent that the estimation domain is strictly restricted to the subsample of general AI chatbot adopters (\(n = 487\)), with caregiving-specific use serving as a conditional binary dependent variable.

3. Methodological Rigor in Qualitative Analysis: Thematic Saturation and Bias

• Comment: While the calculation of percent agreement among the three raters (Table 3) is methodologically sound, the initial generation of the deductive thematic framework was executed by a single investigator (JM). This exposed the exploratory design to potential vulnerabilities of confirmation bias during its inception phase.

• Assessment of Revision: The authors have constructively addressed this by integrating this limitation into lines 465–467. Formally recommending that subsequent implementations utilize independent double-blind thematic coding from inception (rather than post-hoc verification) safeguards psychometric validity and enriches the methodological discussion of the paper.

4. Econometric Specification, Sampling Weights, and Sample Composition

• Comment: Given the stratified, probability-based Internet panel design characteristic of the Understanding America Study (UAS) architecture, standard ordinary least squares or unweighted logit estimations ran the risk of yielding biased standard errors due to heteroscedasticity or spatial/demographic clustering.

• Assessment of Revision: The authors' econometric justification is methodologically impeccable. I agree that the primary objective of Table 4 is to model conditional associations between individual traits, contextual factors, and chatbot use, rather than to produce population-representative descriptive statistics. For this purpose, unweighted regression is standard practice (Solon, Haider & Wooldridge, 2015). Furthermore, the explanation regarding the invalidity of applying general UAS base panel weights to a doubly-selected subsample (caregivers who are also AI users) is technically accurate and avoids introducing artificial bias.

• Language Adjustment: It is highly commendable that the authors carefully revised their language throughout the manuscript, moving away from describing the sample as "statistically representative of the caregiver population" to accurately defining it as "demographically diverse and drawn from a probability-based panel." Additionally, explicitly noting the use of heteroskedasticity-robust standard errors in Table 4 and the narrative text appropriately formalizes the model's statistical inference.

5. Reference Metadata Standardization

• Assessment of Revision: It is confirmed that online datasets and web-based publications (e.g., Pew Research, Ref 10; and Angrisani et al., Ref 11) have been fully revised in strict compliance with PLOS ONE formatting guidelines, appending complete URLs, exact retrieval dates, and permanent traceability parameters.

________________________________________

Recommendation

The current manuscript fully satisfies the standards of empirical rigor, analytical transparency, and mixed-methods structural design required by PLOS ONE. The authors have demonstrated excellent responsiveness to previous evaluations, successfully refining critical areas of econometric modeling and qualitative interpretation. I enthusiastically recommend the manuscript for final acceptance and publication.

Reviewer #2: The manuscript is well-structured, clear, scientifically rigorous, and already incorporates substantial author revisions. I would like to suggest the following comments

1. Use inline bracketed numbers (modified Vancouver style) rather than superscripts.

2. Replace "Insert Figure 1" / "Insert Figure 2" with the formal Fig. 1 and Fig. 2 form.

3. Audit the reference list to clean up redundant references (e.g., refs 11 & 24).

**********

what does this mean?). If published, this will include your full peer review and any attached files.

If you choose “no”, your identity will remain anonymous but your review may still be made public.

**Do you want your identity to be public for this peer review?**  For information about this choice, including consent withdrawal, please see our Privacy Policy

Reviewer #1: No

Reviewer #2: No

**********

# Editor report: Acceptance letter

**DOI:** 10.1371/journal.pone.0357753.r006

**Authors:** Yi Ding

PONE-D-26-08410R2

PLOS One

Dear Dr. Monin,

I'm pleased to inform you that your manuscript has been deemed suitable for publication in PLOS One. Congratulations! Your manuscript is now being handed over to our production team.

At this stage, our production department will prepare your paper for publication. This includes ensuring the following:

* All references, tables, and figures are properly cited

* All relevant supporting information is included in the manuscript submission,

* There are no issues that prevent the paper from being properly typeset

You will receive further instructions from the production team, including instructions on how to review your proof when it is ready. Please keep in mind that we are working through a large volume of accepted articles, so please give us a few days to review your paper and let you know the next and final steps.

Lastly, if your institution or institutions have a press office, please let them know about your upcoming paper now to help maximize its impact. If they'll be preparing press materials, please inform our press team within the next 48 hours. Your manuscript will remain under strict press embargo until 2 pm Eastern Time on the date of publication. For more information, please contact onepress@plos.org.

You will receive an invoice from PLOS for your publication fee after your manuscript has reached the completed accept phase. If you receive an email requesting payment before acceptance or for any other service, this may be a phishing scheme. Learn how to identify phishing emails and protect your accounts at https://explore.plos.org/phishing.

If we can help with anything else, please email us at customercare@plos.org.

Thank you for submitting your work to PLOS One and supporting open access.

Kind regards,

PLOS One Editorial Office Staff

on behalf of

Professor Yi Ding

Academic Editor

PLOS One
